# Why Australia was not wet during spring 2020 despite La Niña

**DOI:** 10.1038/s41598-021-97690-w

**Published:** 2021-09-16

**Authors:** Eun-Pa Lim, Debra Hudson, Matthew C. Wheeler, Andrew G. Marshall, Andrew King, Hongyan Zhu, Harry H. Hendon, Catherine de Burgh-Day, Blair Trewin, Morwenna Griffiths, Avijeet Ramchurn, Griffith Young

**Affiliations:** 1 Bureau of Meteorology, Melbourne, VIC Australia; 2 School of Geography, Earth, and Atmospheric Sciences and ARC Centre of Excellence for Climate Extremes, University of Melbourne, Parkville, VIC Australia; 3 School of Earth Atmosphere and Environment, Monash University, Clayton, VIC Australia

**Keywords:** Climate sciences, Atmospheric science, Atmospheric dynamics

## Abstract

The austral spring climate of 2020 was characterised by the occurrence of La Niña, which is the most predictable climate driver of Australian springtime rainfall. Consistent with this La Niña, the Bureau of Meteorology’s dynamical sub-seasonal to seasonal forecast system, ACCESS-S1, made highly confident predictions of wetter-than-normal conditions over central and eastern Australia for spring when initialised in July 2020 and thereafter. However, many areas of Australia received near average to severely below average rainfall, particularly during November. Possible causes of the deviation of rainfall from its historical response to La Niña and causes of the forecast error are explored with observational and reanalysis data for the period 1979–2020 and real-time forecasts of ACCESS-S1 initialised in July to November 2020. Several compounding factors were identified as key contributors to the drier-than-anticipated spring conditions. Although the ocean surface to the north of Australia was warmer than normal, which would have acted to promote rainfall over northern Australia, it was not as warm as expected from its historical relationship with La Niña and its long-term warming trend. Moreover, a negative phase of the Indian Ocean Dipole mode, which typically acts to increase spring rainfall in southern Australia, decayed earlier than normal in October. Finally, the Madden–Julian Oscillation activity over the equatorial Indian Ocean acted to suppress rainfall across northern and eastern Australia during November. While ACCESS-S1 accurately predicted the strength of La Niña over the Niño3.4 region, it over-predicted the ocean warming to the north of Australia and under-predicted the strength of the November MJO event, leading to an over-prediction of the Australian spring rainfall and especially the November-mean rainfall.

## Introduction

Australian climate is the most predictable in springtime when large-scale oceanic and atmospheric circulations typically exhibit large swings—the El Niño-Southern Oscillation (ENSO) is usually in its growth phase^1,2^; the Indian Ocean Dipole (IOD) anomalies typically peak^1,3^ with or without a relation to ENSO^4,5^; the Antarctic stratospheric polar vortex (SPV) anomaly usually peaks^6–8^; and the Southern Annular Mode (SAM)^9,10^ is strongly influenced by ENSO and the Antarctic SPV^8,11,12^. These climate drivers play important roles in promoting climate anomalies over different parts of Australia^4,13^, overall providing enhanced predictability across northern and eastern Australia compared to the long-term mean behaviour (i.e., climatological forecast) or persistence from the preceding conditions.

The 2019 spring to early summer climate was a good example of high predictability of Australian climate: the IOD was near-record positive and was complemented by a central Pacific El Niño event, the Antarctic SPV weakened dramatically becoming the near-weakest on record, and the October-December mean SAM was the most negative on record for that season in the last 40 years^14,15^. Consequently, many regions of Australia during spring and early summer 2019 experienced record hot and dry conditions especially over the Murray-Darling basin and along the east coast^15,16^, which promoted severe bushfires that caused loss of lives and properties^15,17^. The Bureau of Meteorology's dynamical sub-seasonal to seasonal forecast system, ACCESS-S1 (Australian Community Climate Earth System Simulator-Seasonal version 1)^18^, skilfully predicted the large-scale conditions and their impacts on Australia a season in advance^14,15,19^ (http://www.bom.gov.au/climate/ahead/outlooks/archive/20190815-outlook.shtml), which was consistent with its well-documented ability to simulate and predict many aspects of Australia's weather and climate during spring and early summer^20–24^.

**Figure 1:**
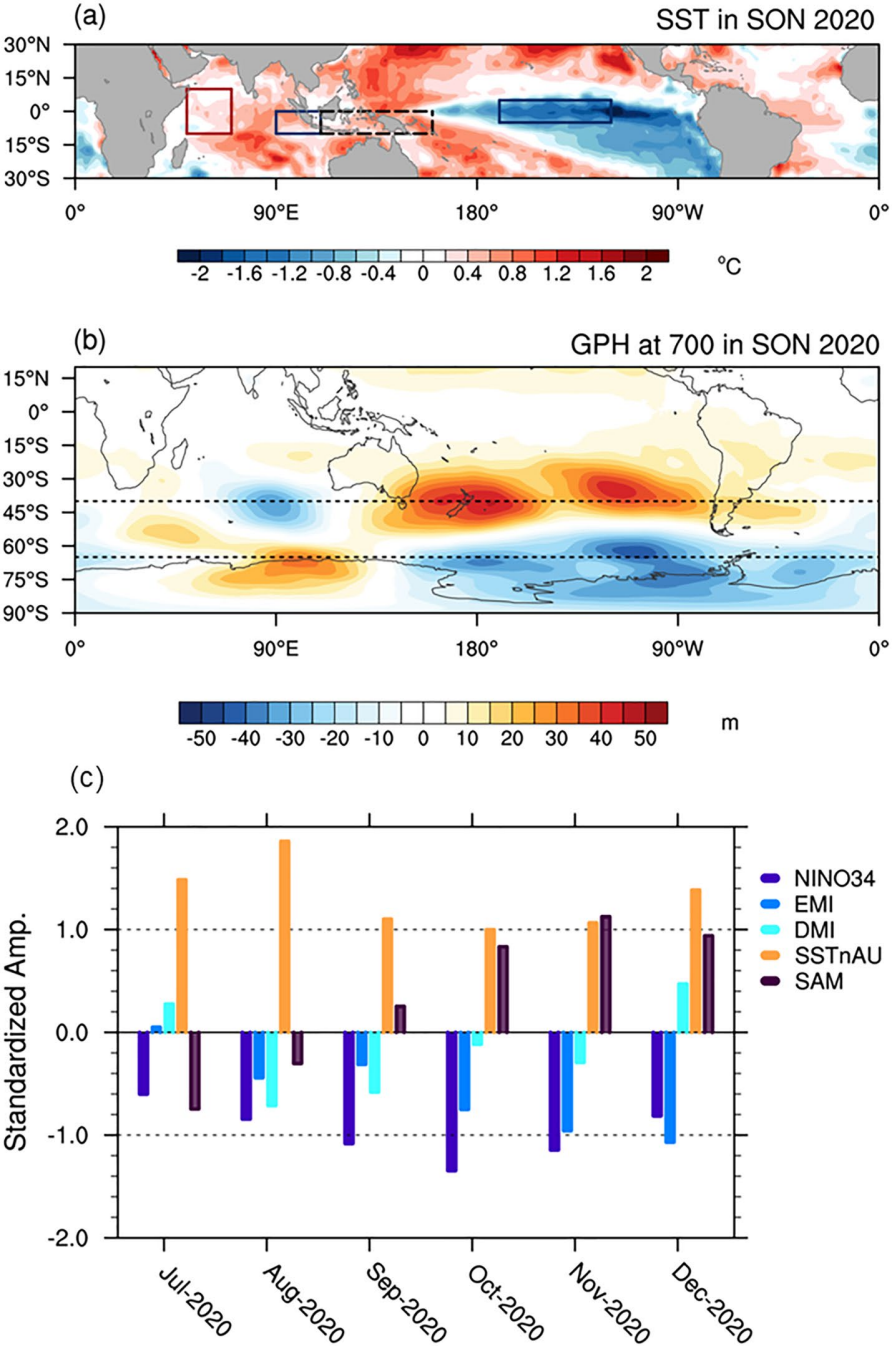
Large-scale conditions of the sea surface and the atmosphere in austral spring 2020. (**a**) September–November mean (SON; i.e., spring) SST anomalies of 2020 relative to 1990–2012 climatology. The red and blue boxed areas are the western pole and the eastern pole of the IOD, respectively; the long-dashed black boxed area is the domain for the SSTs north of Australia considered in this study; and the blue boxed area over the tropical Pacific shows the Niño3.4 region. (**b**) 700-hPa geopotential height anomalies of SON 2020. The short dashed horizontal lines show 40°S and 65°S where nearly zonally symmetric meridional dipole of the SAM is usually found. (**c**) Oceanic and atmospheric climate indices during the second half of 2020. The dark blue, light blue, aqua blue, orange and purple bars indicate the monthly mean values of the Niño3.4 SST, the El Niño Modoki index^33^, the Indian Ocean Dipole mode (IOD) index^3^, the SSTs north of Australia, and the SAM index^9^, respectively. Details of the climate indices shown in (**c**) are described in “Methods”. Maps were generated using the NCAR Command Language version 6.6.2 (www.ncl.ucar.edu).

In contrast, spring 2020 showed much reduced prediction skill for Australia. From mid-2020 onwards La Niña and the negative phase of the IOD were predicted by international forecast models (e.g., https://iri.columbia.edu/our-expertise/climate/forecasts/enso/2020-June-quick-look/?enso_tab=enso-sst_table), and a positive phase of the SAM was also anticipated during austral spring partly due to its relationship with La Niña^11,25,26^. As La Niña, negative IOD and positive SAM are usually associated with springtime wet conditions over different regions of Australia^13,27^, a much-wetter than normal spring was predicted by ACCESS-S1 with high confidence. Both forecasts for La Niña and the positive SAM were verified well, the latter being partly thanks to an extra forcing from the sudden stratospheric polar vortex strengthening during October–November (Fig. 1 and Supplementary Fig. S1a). The negative IOD reached its maximum strength in August (−0.7 standard deviation (σ) anomaly) but then rapidly decayed in October (Fig. 1c).

**Figure 2:**
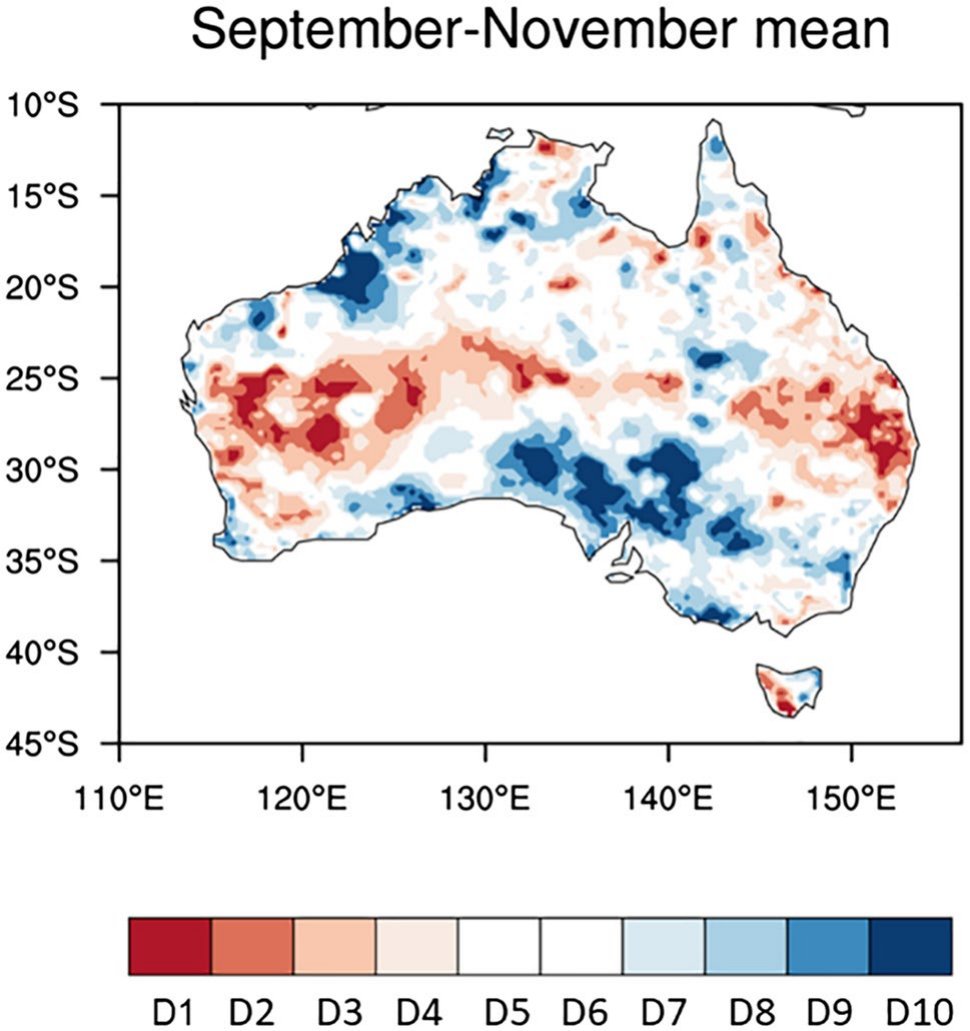
Rainfall decile map of spring 2020. The decile thresholds were obtained from the rainfall data over 1990–2012. A similar decile map but with the thresholds obtained from the full AWAP rainfall record since 1900 is available at http://www.bom.gov.au/climate/maps/rainfall/?variable=rainfall&map=decile&period=3month&region=nat&year=2020&month=11&day=30. The map was generated using the NCAR Command Language version 6.6.2 (www.ncl.ucar.edu).

With these large-scale drivers at play, some parts of Australia received above-average spring rainfall, such as South Australia and northwest Western Australia, but it was markedly less than expected from the emphatically wet forecast, and there was significantly below average rainfall over south-eastern Queensland and north-eastern New South Wales (Fig. 2). This forecast error over eastern Australia was an unpleasant surprise to the users of seasonal forecasts, damaging the credibility of seasonal climate forecasts. In this study we review the large-scale climate features of austral spring 2020 and investigate possible causes of the deviation of rainfall from its historical response to La Niña and causes of the forecast failure.

For the observational analyses over the period 1979–2020 we used: Reynolds OI v2 sea surface temperature (SST) data^28^ for 1982–2020 and Hurrell et al.^29^ SST data for 1979–1981; NOAA interpolated outgoing longwave radiation (OLR) data^30^; Japanese 55-year Reanalysis (JRA-55) data^31^ for the global analysis of atmospheric variables; and the Australian Water Availability Project (AWAP) monthly mean rainfall data over Australia^32^. For the forecast analyses we assessed the ACCESS-S1 forecasts initialised in July to November. For each variable, the observed and forecast climatologies and standard deviations were computed for the period 1990–2012, over which ACCESS-S1 hindcasts are available. Details of statistical analysis methods and ACCESS-S1 system configurations are provided in “Methods”.

## Large-scale climate features and Australian rainfall in spring 2020

SSTs over the Niño 3.4 region (blue box in the tropical Pacific in Fig. 1a) started cooling from mid-2020 and reached minus 1σ anomaly in September 2020 (Fig. 1c). This La Niña developed a central Pacific-flavour with the maximum cold SST anomaly shifting westwards as the season progressed, which is evidenced by amplification of the negative El Niño Modoki Index (EMI)^33^ while the Niño 3.4 SST cold anomaly weakened after October (Fig. 1c). The strength of La Niña of austral spring 2020 was −1.2σ, which was comparable to that of 1998, 1999 and 2007, but weaker than that of 2010 (−1.6 σ) and 1988 (−1.9 σ) as judged by the Niño 3.4 September to November mean SST anomaly.

The local SSTs surrounding Australia were significantly higher than normal, which we attribute to the occurrence of La Niña during the second half of 2020 and the long-term warming trend, which is statistically significant at the 5% level^34,35^. Hendon et al.^13^ demonstrated that northern Australian rainfall in spring significantly increases with higher SSTs north of Australia, which is monitored by an index of the SSTs averaged over the equator-10°S and 110–160°E (SSTnAU; long-dashed black box in Fig. 1a). In austral spring 2020, SSTnAU was about 1σ higher than normal, but this was substantially less than what was expected from the occurrence of the 2020 La Niña and the long-term warming trend of the SSTnAU based on the historical relationships over 1979–2019. Based on these relationships, the expected 2020 value was 2σ above normal, which would have been the warmest on record since 1979 (Supplementary Fig. S2).

The IOD rapidly transitioned from being strongly positive in June (1.6σ), with higher-than-normal SST over its western pole and lower-than-normal SST over its eastern pole (red and blue boxes in Fig. 1a), to being moderately negative (−0.7σ) in August. The negative IOD did not last through austral spring but decayed in October (Fig. 1c). Consequently, the springtime mean IOD amplitude was only −0.3σ. A more pronounced feature over the tropical Indian Ocean was anomalous warming in the central part of the basin, which perhaps was contributed to by the long-term upward trend^34,35^. Over the period of 1979–2019, there has been a positive trend in the IOD index (Dipole Mode Index; DMI), statistically significant at the 10% level in spring^36,37^. Once the linear trend was removed, the springtime mean IOD amplitude was -0.7σ, which implies that the long-term warming trend in the tropical Indian Ocean acted to weaken the temperature difference between the east and west poles of the negative IOD in spring 2020.

The SAM was in its positive phase, exhibiting moderate strength in October-December (Fig. 1c). However, the near-surface circulation anomalies during spring 2020 did not follow a canonical zonally symmetric meridional dipole pattern that has a mid-latitude zonal wavenumber-3 feature embedded^11^. Instead, its zonal symmetry was disrupted by an equivalent barotropic Rossby wave train emerging from the tropical Indian Ocean, leading to the zonal wavenumber-1 pattern in the SH high latitudes (Fig. 1b). This wave pattern highlights that the anomalous tropical central Indian Ocean warming mentioned above is likely to have exerted strong forcing on the circulation over the Southern Indian Ocean. The promotion of the positive SAM in late spring of 2020 could partly be attributed to La Niña^26^, but is probably more closely related to the downward coupling from the near-record strengthening of the Antarctic stratospheric polar vortex that occurred in October and November 2020, which developed in conjunction with an extraordinary lack of upward propagation of planetary-scale waves from the troposphere during spring 2020 (https://ozonewatch.gsfc.nasa.gov/meteorology/figures/merra2/heat_flux/vt1-3w45_75-45s_100_2021_merra2.pdf) (Supplementary Fig. S3).

**Figure 3:**
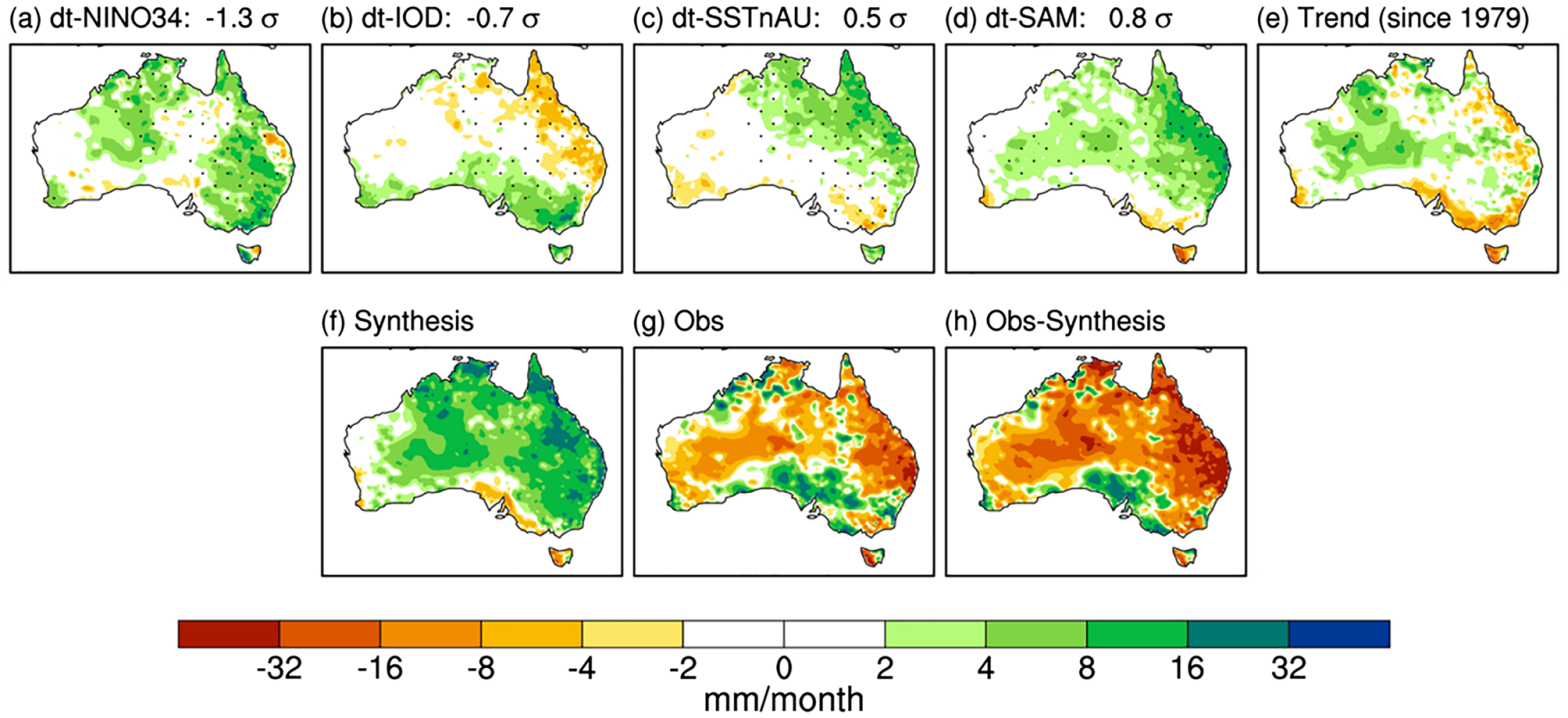
Synthesis of 2020 spring rainfall anomalies using multiple linear regression onto key climate indices. The contributions from the individual predictors are (**a**) de-trended Niño3.4 SST index, (**b**) de-trended DMI, (**c**) de-trended SSTs north of Australia (eq-10°S,110–160°E), (**d**) de-trended SAM, and (**e**) time (i.e., trend). The full synthesis using all five predictors is shown in (**f**). (**g**) Observed 2020 spring rainfall anomaly relative to the climatology of 1990–2012. (**h**) Difference between the observed and the synthesized rainfall. The BoM official base period is 1961–1990, and the anomaly pattern relative to this earlier base period is similar to (**g**) (http://www.bom.gov.au/climate/maps/rainfall/?variable=rainfall&map=anomaly&period=3month&region=nat&year=2020&month=11&day=30). In (**a**)–(**e**) synthesis anomalies were computed by the regression coefficients obtained over 1979–2019 and then scaled by the predictor values of 2020 (see “Methods”). Stippling in (**a**)–(**e**) indicates where correlation between the rainfall and each predictor is statistically significant over 1979–2019 at the 5% level. Maps were generated using the NCAR Command Language version 6.6.2 (www.ncl.ucar.edu).

A regression analysis (Fig. 3 and Supplementary Figs. S4, S5) of the historical relationship of Australian springtime rainfall with these well-known large-scale oceanic and atmospheric springtime seasonal climate drivers—ENSO, SSTnAU, IOD and SAM— and a 40-year trend produced a strong prediction of a wet spring for 2020 (Fig. 3f) despite the mixed contribution from the weak negative IOD (Fig. 3b). In other words, a statistical seasonal forecast based on the occurrence of these key drivers anticipated a very wet spring.

**Figure 4:**
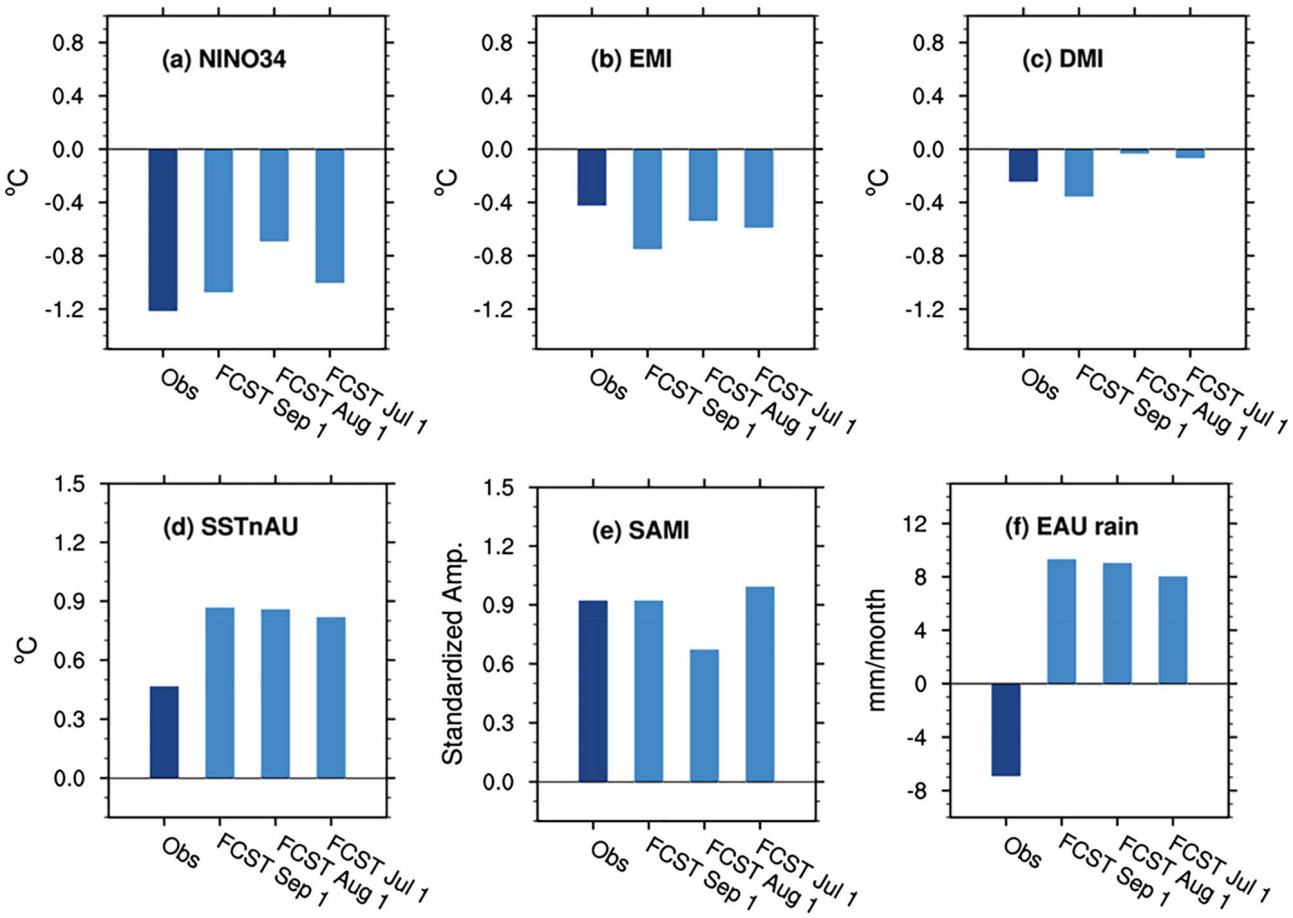
Observed and forecast climate indices and eastern Australian rainfall for spring 2020 (September–November mean). The ACCESS-S1 99-member forecasts were initialised on 1 July, 1 August, and 1 September (light blue bars) (see “Methods”for the details of the formation of the ensemble). The dark blue bars display observed values. In (**f**) observed and forecast rainfall was averaged over eastern Australia (east of 140°E; EAU rain).

The Bureau of Meteorology’s dynamical forecast system ACCESS-S1^18^ skilfully predicted the occurrence of La Niña in austral spring as judged by the forecast Niño3.4 SST and the associated spatial details of the tropical SST anomalies as depicted by the negative EMI and positive SSTnAU with up to two months lead time (Fig. 4a, b, d). However, the local SSTs north of Australia were predicted to be too warm, with the predicted positive anomaly being almost two times greater than observed, when the forecasts were initialised on 1 July, 1 August, and 1 September (Fig. 4d). On the other hand, ACCESS-S1 predicted the weak negative IOD of spring only at zero lead time (i.e., initialised on 1 September), having predicted neutral IOD conditions in earlier forecasts (Fig. 4c). Positive SAM was correctly predicted from the beginning of July (Fig. 4e) likely due to its connection with La Niña in the forecast because the anomalous strengthening of the stratospheric polar vortex in October–November 2020 was not predictable until late September (Supplementary Figs. S1b and S6).

**Figure 5:**
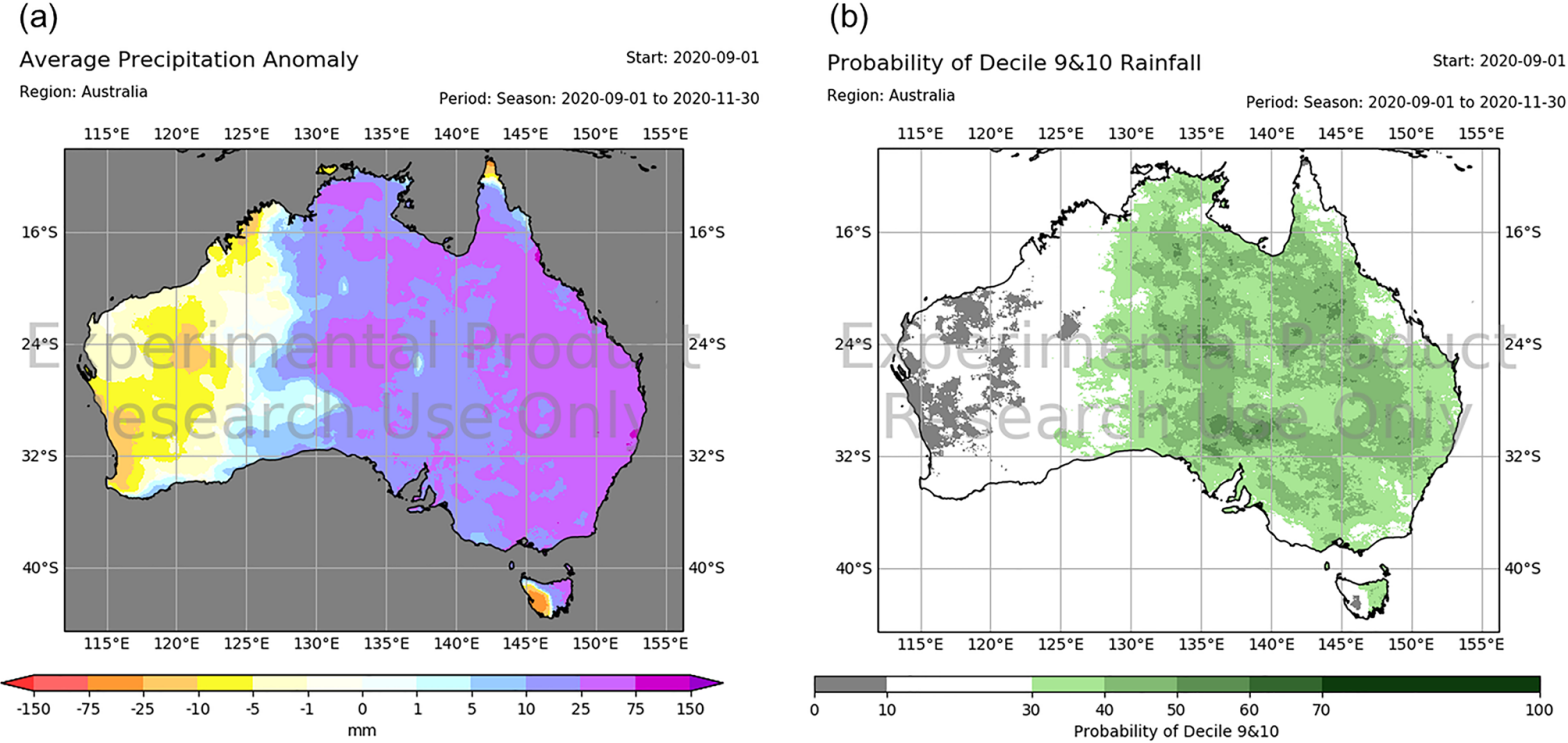
ACCESS-S1 99-member forecasts for spring rainfall, initialised on 1 September 2020. (**a**) Rainfall anomaly and (**b**) probability of Decile 9 and 10 (i.e., top 20% category). Maps were generated with Python version 3.6.1 (https://www.python.org/).

Together with the forecasts of tropical Indo-Pacific SST conditions and positive SAM, spring rainfall over central and eastern Australia was predicted to be significantly higher than normal from the initial conditions of July onwards (Fig. 4f). These dynamical forecasts are broadly consistent with the statistical forecast based on the historical relationships presented in Fig. 3. Even at zero lead time, ACCESS-S1 predicted eastern Australia to be significantly wetter than average, and the forecast probability for spring rainfall in the top 20% category (based on the hindcast period 1990–2012) was significantly higher than the climatological probability (i.e., > 20%) (Fig. 5). Further analysis using 99-member ensemble forecasts indicates that the rainfall amount of individual forecast members over eastern Australia was strongly determined by their SSTnAU forecasts (correlation (*r)* > 0.4 at lead times 0 and 1 month) and, to a lesser degree, by the EMI and SAM forecasts (*r* > 0.2 at lead times 0 and 1 month) (Supplementary Fig. S7).

**Figure 6:**
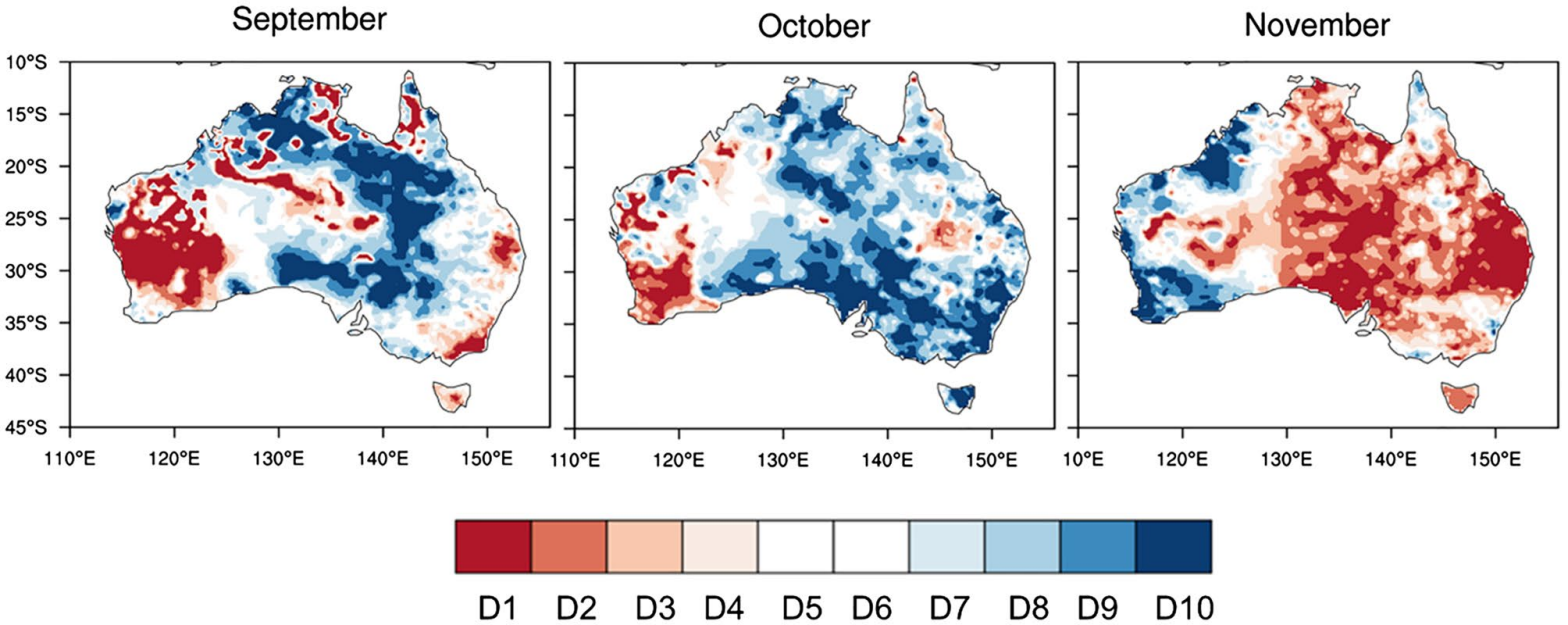
Rainfall decile maps of austral spring months. The decile thresholds were obtained from the period 1990–2012. Similar decile maps but based on the thresholds obtained from the full AWAP rainfall record since 1900 are available at http://www.bom.gov.au/climate/maps/rainfall/?variable=rainfall&map=decile&period=month&region=nat&year=2020&month=11&day=30. Maps were generated using the NCAR Command Language version 6.6.2 (www.ncl.ucar.edu).

**Figure 7:**
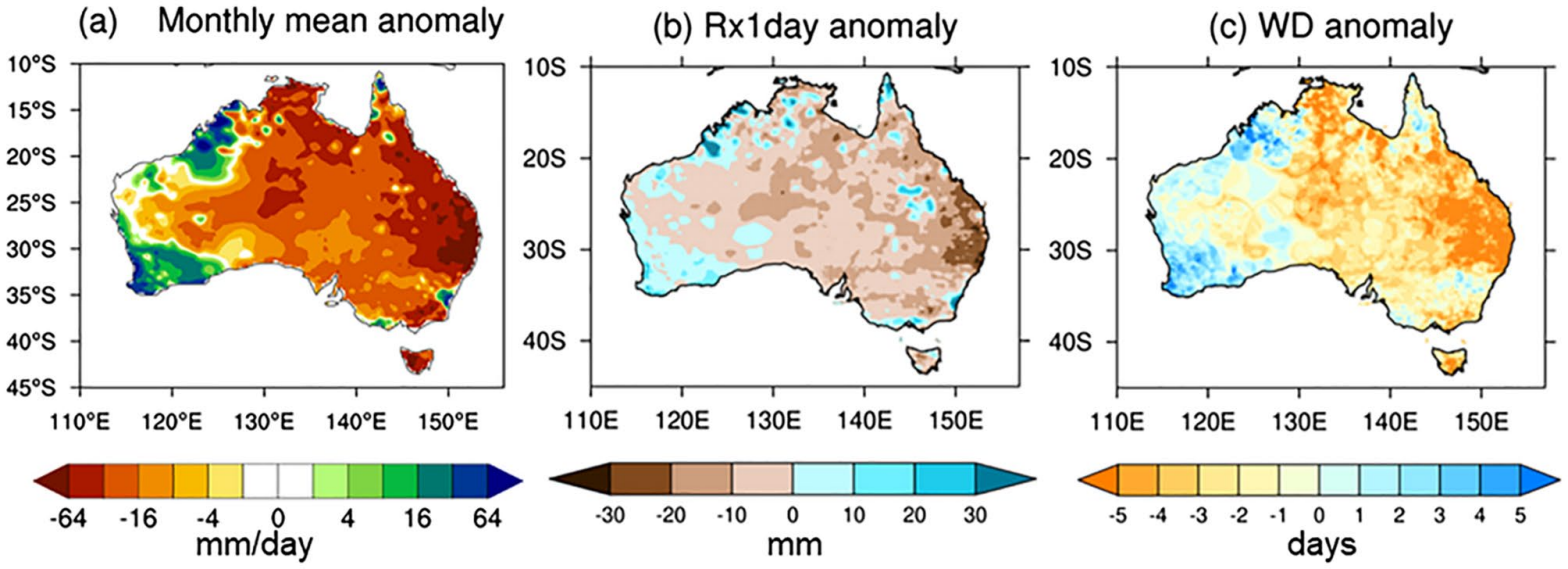
November 2020 rainfall anomalies. (**a**) Monthly mean, (**b**) 1-day maximum rainfall amount of the month (Rx1day; i.e., the intensity of the wettest day in the month), and (**c**) number of wet days (> 1 mm; WD). Maps were generated using the NCAR Command Language version 6.6.2 (www.ncl.ucar.edu) (**a**) and the IDL version 8.6 (https://idlsgroup.com/) (**b**,**c**).

Despite all these strong reasons to anticipate a wet spring, much less rain fell than was expected—more than half of the country turned out to be drier than average (Fig. 3g) (relative to the mean climate of 1990–2012 in this study, but this is also the case relative to the earlier period mean climate of 1961–1990; http://www.bom.gov.au/climate/maps/rainfall/?variable=rainfall&map=anomaly&period=3month&region=nat&year=2020&month=11&day=30). Figure 2 shows that the spring rainfall deficit was particularly severe, being in the bottom 10–20% of the climatological period of 1990–2012 in some locations of far eastern and south-eastern Queensland where excessive rainfall was confidently predicted. Such a dry season was predominantly contributed to by significantly below-normal rainfall in November 2020 (Figs. 6, 7a) when extreme dry conditions (i.e., bottom 10%) occurred in all states and territories of Australia (Fig. 6), although the rainfall in September and October was also less than expected (Fig. S8). In particular, there was a significant reduction in the number of rainy days (i.e., rainfall > 1 mm), which explains the extreme dry conditions in the Northern Territory and southern Queensland with the latter location also being impacted by below-average maximum daily rainfall for the month (Fig. 7b, c). So, why was November so dry in the midst of strong La Niña and associated SST conditions that were favourable for a wet spring over eastern Australia?

## November dryness over Australia

**Figure 8:**
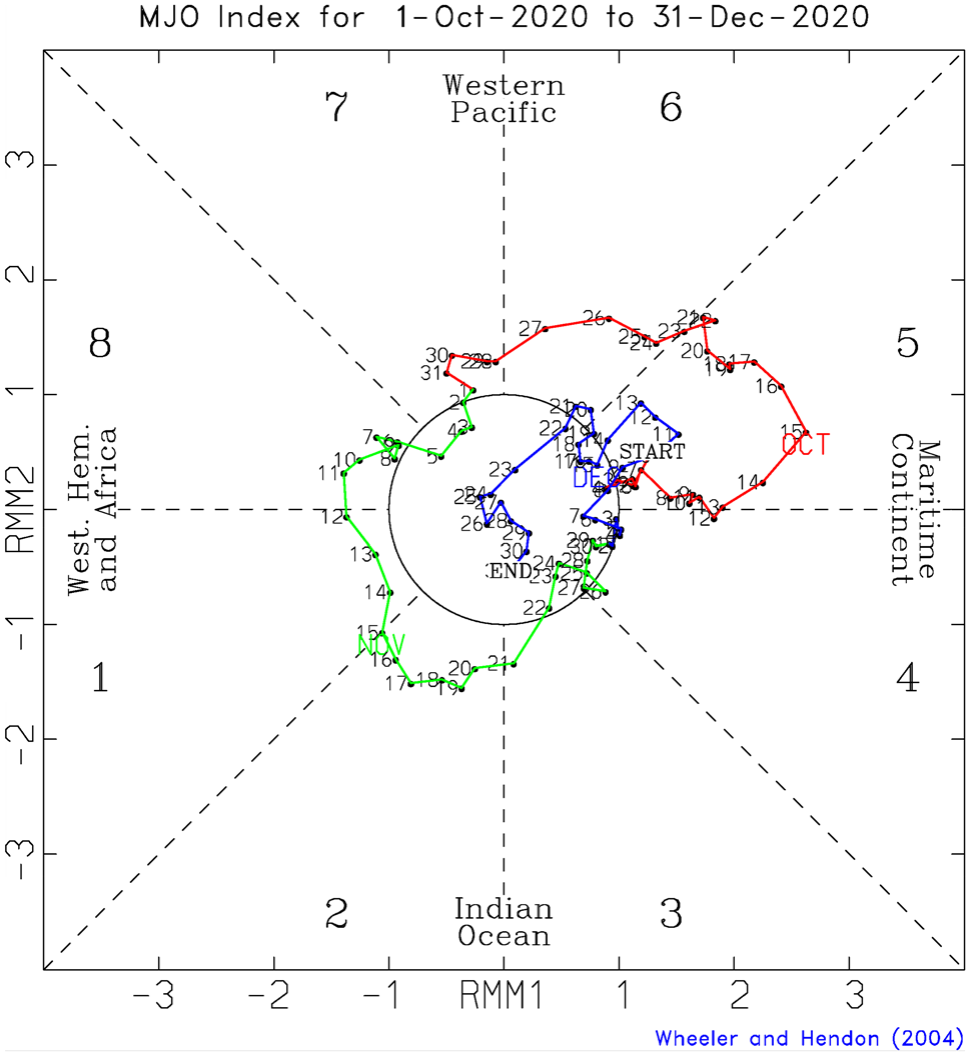
Madden–Julian Oscillation (MJO) in October-December 2020. Phase space of the Real-time Multivariate MJO (RMM) index of Wheeler and Hendon (2004)^40^ for the period 1 October to 31 December 2020. Each dot represents a day and the sequence of days is joined by coloured lines. In October (red) the MJO was primarily in Phases 5 and 6, in November (green) it was primarily in Phases 8, 1, 2, and 3, and in December (blue) in Phase 5 and the Weak MJO circle.

In addition to the dominant seasonal modes of variability described above, another key driver of Australian weather and climate variability, the Madden–Julian Oscillation (MJO)^38^, was active on the sub-seasonal time scale through spring 2020. The convective phase of the MJO passed over the Maritime Continent and the equatorial western Pacific in October and over the western hemisphere, Africa, and western-central Indian Ocean in November 2020 (Fig. 8). Previous work exploring MJO teleconnections to Australian weekly climate anomalies in each season has shown that during September–November, drier-than-normal conditions occur over eastern and south-eastern Australia when the MJO convective activity is enhanced over the central Indian Ocean and suppressed over the far western Pacific. This is depicted as the MJO being in Phases 2 and 3 as displayed at http://www.bom.gov.au/climate/mjo/#tabs=Average-conditions^39^.

**Figure 9:**
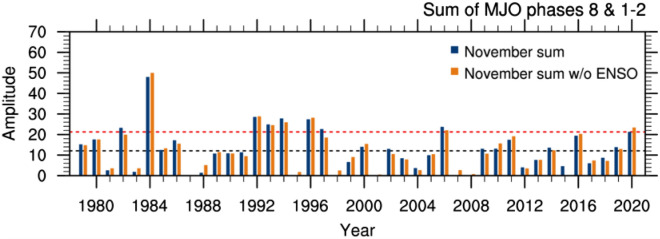
Time series of the sum of the daily MJO amplitudes for Phases 8, 1 and 2 during November (referred to as MJO812). MJO amplitudes in Phases 8, 1, and 2 were summed only if they were equal to or greater than 1 over more than one day in the month. The blue bars show the sum of the daily raw amplitudes for the occurrence of Phases 8, 1, and 2, and the orange bars show the same but with the linear dependence on the Niño 3.4 SST index removed. The black and orange coloured horizontal dashed lines indicate the mean and the positive 1 standard deviation of the ENSO-free MJO812 time series from its mean, respectively.

In November 2020, the MJO propagated through Phases 7, 8, 1, 2, 3, and 4, with the most time and strongest amplitudes in Phases 8, 1, and 2. As a novel attempt to capture the impact of the MJO on the monthly total rainfall in November 2020, we formed an index for November by summing the daily amplitudes of the MJO in Phases 8, 1 and 2 (MJO812) whenever they were equal to or greater than one for more than one day in each phase. We used the amplitude of the MJO based on the Real-Time Multivariate MJO (RMM) Index available at the BoM^40^ (see “Methods”). In forming the MJO812 index, we removed the ENSO signal by linearly regressing it out from the raw MJO812 index. Because the interannual variability was already removed from the RMM indices^40^, the raw November MJO812 index is not statistically significantly correlated with the Niño3.4 SST index at the 10% level, assessed by a two-tailed Student^41^ t-test with the 41 samples of 1979–2019. Nevertheless, some of the high MJO812 activities occurred during strong El Niño years such as 1982 and 1997. Therefore, removing the ENSO signal from the MJO812 index was appropriate to select the ENSO-free high MJO events. The raw and the ENSO-removed MJO812 time series for November are shown in Fig. 9. The amplitude of the MJO812 anomaly during 2020 was about 1σ. Without the ENSO-related component, the November 2020 value was the 6^th^ strongest in the recent 42-year record.

To understand the impact of high MJO812, we have made the composite differences of November-mean outgoing long wave radiation (OLR) and Australian rainfall between the 6 highest amplitude MJO812 years (1984, 1992, 1993, 1994, 1996, 2006) and the 8 lowest amplitude MJO812 years (1987, 1995, 1998, 2001, 2004, 2007, 2008, 2015). The years were selected based on the ENSO-free MJO812 index in Fig. 9 being greater than |1σ|. The influence of ENSO on November-mean OLR flux and rainfall was also removed by regression before making the composite differences.

**Figure 10:**
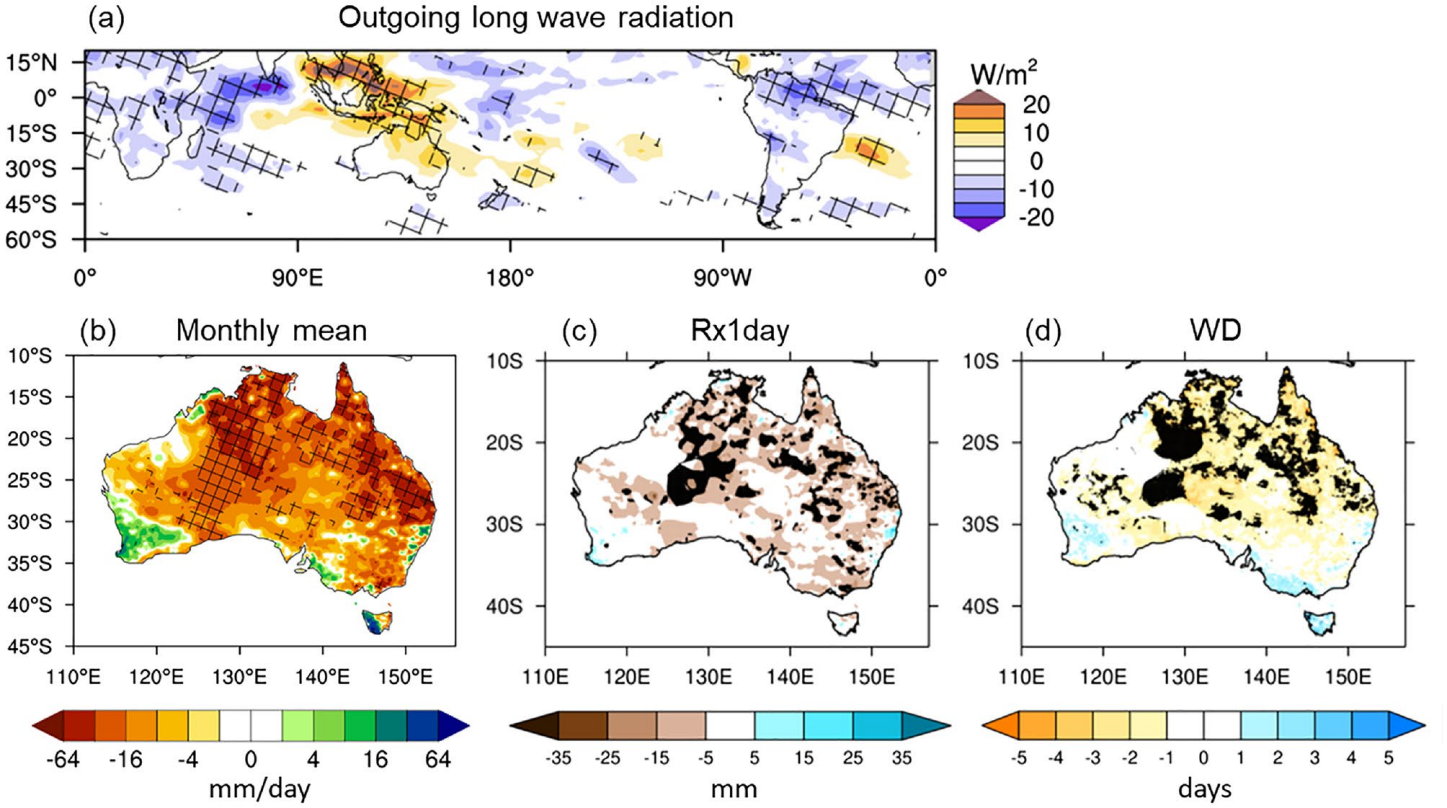
Composite difference between 6 highest and 8 lowest MJO812 indices during November. (**a**) Mean outgoing long wave radiation (OLR), (**b**) monthly mean rainfall, (**c**) 1-day maximum rainfall amount, and (**d**) number of wet days. Before forming the composites, the ENSO-related components were removed by regressing out the linear dependence on the Niño3.4 SST index. The hatched areas in (**a**) and (**b**) and the black coloured areas in (**c**) and (**d**) indicate statistically significant differences at the 5% level. In (**c**) and (**d**), the black shaded differences in central Australia are unlikely to be a robust feature because it is a very dry area, and therefore, differences could be dominated by one or two events, particularly given the small sample sizes in the two MJO groups. Maps were generated using the NCAR Command Language version 6.6.2 (www.ncl.ucar.edu) (**a**,**b**) and the IDL version 8.6 (https://idlsgroup.com/) (**c**,**d**).

High values of the MJO812 index are historically associated with November-mean enhancement of convection over the western to central equatorial Indian Ocean and suppression of convection over the eastern Indian Ocean and the Maritime Continent (Fig. 10a). When MJO812 is high during November, the western half of the Northern Territory/the eastern half of Western Australia and eastern Queensland, especially the southeast corner of Queensland, experience extensive dryness compared to when MJO812 is anomalously low (Fig. 10b). Strikingly, this rainfall pattern associated with high MJO activity in Phases 8, 1 and 2 matches well with the extreme rainfall deficit during November 2020 (Figs. 6 and 7a), suggesting that the MJO activity over the Indian Ocean and its teleconnection played a key role in acting to dry eastern Australia after the negative IOD decayed. Composite differences of 1-day maximum rainfall amount and the number of wet days suggest that the number of rainy days reduces over northern and north-eastern Australia when MJO812 is anomalously high, and both number of rainy days and 1-day maximum rainfall amount significantly reduce over the southeast corner of Queensland, where the dry response of the November mean rainfall to MJO812 is especially strong (Fig. 10c, d).

For the hindcast period of 1990–2012, ACCESS-S1 demonstrates skill in predicting the MJO, based on the RMM indices, out to about four weeks lead time^42^. Seasonally, this skill extends to 30 days in austral winter and spring and reduces slightly to 25 days in austral summer and autumn^23^. Together with this predictive skill of the MJO, ACCESS-S1 also captures the observed modulation of extreme rainfall by the MJO in weeks two and three of the forecast^23^. This translates to enhanced skill for predicting MJO-related extreme rainfall across much of Australia during spring and summer^23^. For instance, some of the largest forecast skill improvements in spring are achieved for northern Australian rainfall during MJO Phase 8 and for central Australian rainfall during MJO Phase 2 when the MJO is strong as compared to when it is weak. Moreover, in each of the MJO Phases 8, 1 and 2 the model shows useful skill (better than a random forecast) for predicting dry extremes over most of Australia including the east (not shown). Thus, if ACCESS-S1 was to adequately predict the state of the MJO in November 2020 in its Phases 8, 1 and 2, it should have provided some indication of the dry conditions over Australia in the forecasts made from the second half of October 2020.

**Figure 11:**
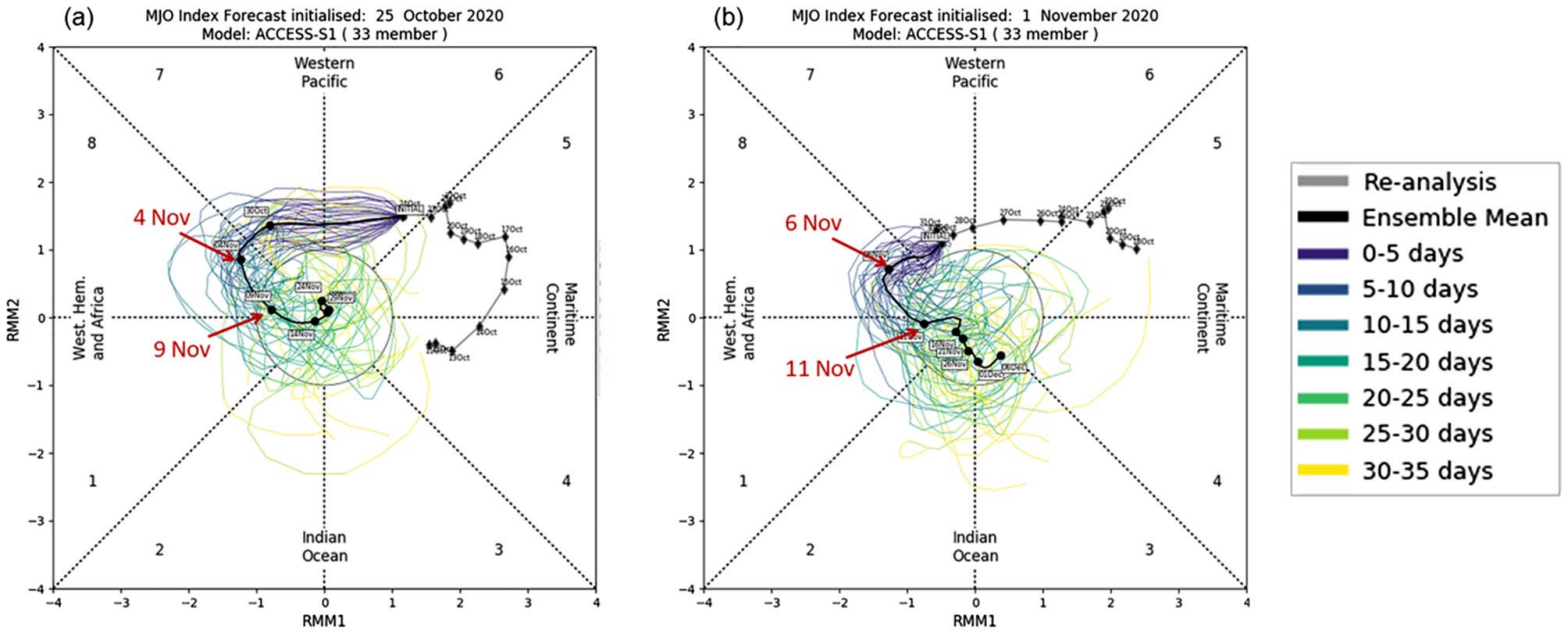
ACCESS-S1 forecasts for the MJO in November 2020. Forecasts were initialised on (**a**) 25 October and (**b**) 1 November of 2020. A 33-member burst ensemble was used for these diagrams. The grey line indicates the observed trajectory of MJO over the 15 days leading up to the forecast initialisation date, and the thick black line indicates the ensemble mean forecast. The thin coloured lines indicate the ensemble member forecasts, and the different colours denote the different verification windows as shown by the legend. The ensemble mean forecasts shown in solid black lines are to be compared to the green line in Fig. 8.

**Figure 12:**
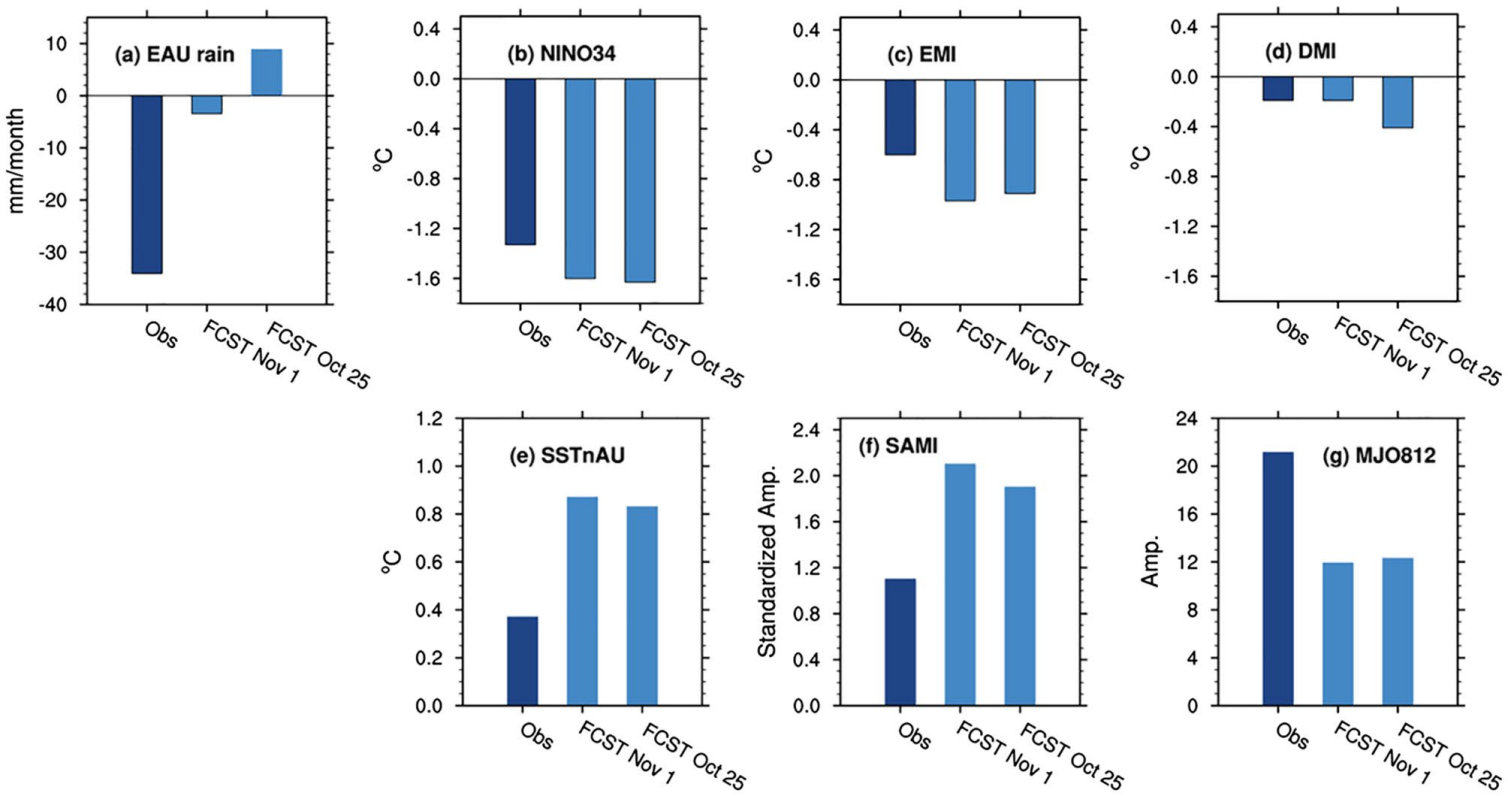
ACCESS-S1 forecasts initialised on 25 October and 1 November 2020 for eastern Australian rainfall and climate indices for November 2020. In (**g**) raw MJO812 (i.e., without removing its association with the Niño3.4 SSTs) is displayed to simplify the comparison with the real-time forecasts.

The ACCESS-S1 ensemble mean forecasts for the MJO initialised in late October onwards were able to predict the amplification of the MJO in Phase 8 in the first 10 days of November (Fig. 11). However, as the spread of the ensemble grew rapidly after that, the ensemble mean forecast MJO rapidly weakened and did not evolve through Phases 1 and 2 during mid- to late November. This failure is likely to stem from the forecast MJO being unable to maintain its suppressed convection over the Maritime Continent and the western Pacific where anomalously enhanced convection was forced by the forecast La Niña. In reality, however, the MJO propagated through that region (Supplementary Fig. S9). Consequently, the ensemble mean forecast for the MJO812 index during November 2020 was only about half the strength of the observed (Fig. 12g). The 33-member ensemble forecasts of northern and eastern Australian rainfall show some sensitivity to the forecast strength of MJO812 when they were initialised on 25 October, but it was much weaker than the observed (Supplementary Fig. S10).

While the MJO812 amplitude and its connection to Australian rainfall were significantly under-predicted, La Niña and the associated westward shift of the maximum cold SST anomaly (as captured by EMI), the SST anomalies to the north of Australia and the negative IOD were all over-predicted even at the shortest lead time for the month of November (except for the IOD at 0 lead time) (Fig. 12b–e). Moreover, ACCESS-S1 significantly over-predicted the positive SAM, with more than doubling of the observed strength that may result from too strong downward coupling from the stronger-than-normal stratospheric polar vortex over Antarctica (Fig. 12f). The forecast of overly strong positive SAM would have further raised the odds of increased rainfall over subtropical Australia and the odds of decreased rainfall over western Tasmania as ACCESS-S1 skilfully simulates the SAM-Australian rainfall connection in late spring when the SAM forcing is strong^15^. Therefore, it appears that a combination of these different factors led to the over-prediction of wet conditions in subtropical Australia for November 2020 (Fig. 12a).

## Summary and concluding remarks

La Niña is well known to be associated with above normal rainfall over Australia and has often acted in the past to terminate antecedent multi-year droughts^13,43–46^. Furthermore, La Niña boasts long-lead predictability, even beyond one year^47^, that stems from the ocean wave dynamics known as the delayed oscillator^48^ or recharge-discharge oscillator^49^, which is expressed at the sea surface as La Niña typically developing after strong El Niño. This long-lead predictability of La Niña implies that the Australian rainfall component associated with La Niña should be predictable at long-lead times. From mid-2020 international seasonal climate forecast models—both statistical and dynamical models alike—predicted the occurrence of La Niña with a moderate strength (0.5–1 °C at the Niño 3.4 region) (https://iri.columbia.edu/our-expertise/climate/enso/) and a significant strength of the negative IOD (http://www.bom.gov.au/climate/model-summary/archive.shtml). Accordingly, Australia was predicted to be wetter than normal for spring 2020 from the forecasts made from winter 2020 right up to the beginning of the spring, and there was a strong consensus among international forecast models. However, this confident forecast for excessive rainfall did not verify well with rainfall being near or below average in many locations, especially over south-eastern Queensland. Incorrect forecasts, such as this for spring 2020, not only have a negative impact on user-confidence in seasonal forecasts but also have significant economic and social repercussions particularly if they come at key decision times. For example, stakeholder feedback suggests that the wet outlook for November 2020 caused producers to rush grain harvests and work excessive hours, resulting in pressure on workers' health and safety to get the crops harvested before it rained (Adrian McCabe, Grain Producers South Australia, pers comm). These types of situations can result in significant mental stress and considerable expense (e.g., having to get in extra labour and equipment to harvest and transport grain). Therefore, in this study we have reviewed the climate features of austral spring 2020 in detail, evaluated forecasts from the BoM’s ACCESS-S1 system, and attempted to understand why Australia was not wet in its spring despite the occurrence of La Niña 2020 and why the forecast system did not predict the abnormal dry response.

During spring 2020, the negative IOD did not strengthen through the season but started decaying from August, which was substantially earlier than the typical life cycle of the IOD^1^. Furthermore, although the SSTs north of Australia were warmer than normal, they were only a half the strength of the expected anomaly given the magnitude of the Niño3.4 SST of spring 2020 and the local linear SST trend since 1979. Because La Niña impacts Australian climate via (1) the strengthened Walker circulation, which is coupled to the warmer-than-normal SSTs in the far western Pacific region, including the SSTs surrounding northern Australia^50,51^ and also (2) via the concurrent negative IOD^4,36,52^, the moderate warming of the local SSTs north of Australia and the early termination of the negative IOD are likely to have contributed to the drier-than-expected spring over many areas of Australia in 2020 despite the presence of La Niña.

Moreover, the drying was pronounced in November, particularly over south-eastern Queensland and central Australia as evidenced by rainfall totals falling in the bottom 20% of the historical record since 1900 in many locations. We found that this dry anomaly was largely due to the strong MJO activity over the tropical Indian Ocean, which was depicted as the 6^th^ strongest event in November by our novel MJO812 index that represents the duration and strength of the MJO in Phases 8, 1 and 2 during the month. The composite differences of Australian November-mean rainfall (as well as the 1-day maximum rainfall amount and the number of wet days) between the high and low amplitude MJO812 years selected from the period 1979–2019 reveal that MJO812 is associated with strongly supressed monthly mean convection over the Maritime Continent and northern and eastern Australia and so can significantly reduce rainfall there in November. This composite difference of November rainfall for high and low MJO812 years matches the November rainfall anomaly of 2020 remarkably well, confirming that the MJO was a key driver of the dry conditions that developed during November 2020.

According to the assessment of the ACCESS-S1 hindcasts^42^, skilful predictions of the MJO are possible out to about four weeks, which means that the November MJO essentially represents unpredictable short time scale "noise" for the seasonal climate forecasts issued at the start of September or earlier. This intrinsic limitation in the predictability of the MJO probably explains why seasonal forecasts from all international models failed to forecast drier-than-normal spring conditions of 2020. However, even when forecasts were initialised in early November, ACCESS-S1 did not successfully predict the large MJO amplitudes in Phases 1 and 2 that were observed in mid-late November 2020. Whether this forecast failure to sustain the eastward propagation of the strong MJO in Phases 1 and 2 reflects a sensitivity to the other forecast atmospheric/sea surface conditions of November 2020 as briefly discussed in the previous section or reflects a systematic bias of ACCESS-S1^53^ needs further investigation.

In contrast to the MJO prediction, ACCESS-S1 substantially over-predicted the positive SST anomalies to the north of Australia for the spring months, which appears to have enhanced a forecast wet signal over northern and eastern Australia. In addition, November SAM was predicted to be too strongly positive, which was likely due to too-strong coupling with the Antarctic stratospheric polar vortex strengthening for forecasts initialised in late October to early November. The under-prediction of the MJO and associated teleconnection might have exacerbated the over-prediction of the positive SAM and SAM-driven rainfall over Australia for November.

In general, seasonal forecast systems have less skill in predicting SSTs outside of the tropical central to eastern Pacific Ocean^54^. Although ACCESS-S1 performs significantly better than climatology or persistence with respect to predicting monthly and seasonal climate variability, there is still much room for improvement. For instance, to improve the forecast accuracy and reliability of rainfall over Australia, the forecast accuracy of the SSTs surrounding Australia, the MJO and the SAM will have to be improved.

Although the impact of decadal variability on the climate of spring 2020 was not considered in this study, it is worth mentioning that the earlier demise of the negative IOD despite the presence of La Niña during spring 2020 may have been influenced by the probable current cold phase of the Inter-decadal Pacific Oscillation (IPO)^55,56^ (Supplementary Fig. S11). In the most recent cold IPO mean state, the IOD and ENSO were found to be more independent of each other in austral spring compared to the previous warm IPO mean state^56^. Carefully designed forecast sensitivity experiments to the ocean initial conditions may shed some light on the impact of the ocean mean state on this IOD event of 2020 and its predictability.

Finally, there is a hint of a long-term drying trend since 1979 over eastern Queensland, the south-eastern Australian coast, western Tasmania, and southwest Western Australia according to Fig. 3e. This drying trend is similar to that in winter, but it is not statistically significant at the 10% level in spring. In contrast, a long-term wetting trend over north-eastern Western Australia in spring is statistically significant at the 5% level, which resembles the summer rainfall trend. It is worth noting that the overall trend pattern bears some resemblance to the projected rainfall change under increasing greenhouse gas emission scenarios using the models of the Coupled Model Inter-comparison Project phase 6 (CMIP6)^57^. These weak to moderate linear trends of rainfall in spring stem, in part, from the long-term trends in different seasonal climate drivers, some of which may oppose each other to result in a smaller impact. For instance, there has been a weak positive trend in the IOD in spring as mentioned earlier, which would act to reduce spring rainfall in the southern part of the country, while there has been a strong positive trend in the SSTs north of Australia, which would act to increase spring rainfall in the northern part of the country. As austral spring is the season when the large-scale climate drivers tend to have big swings in their positive and negative phases and significantly impact Australian seasonal climate, better understanding the mechanisms of these large-scale modes of climate variability, their interactions with one another, and their representation in climate prediction models will be key to understanding future changes in Australian sub-seasonal to seasonal climate and its predictability.

## Methods

### Climate indices

In this study, ENSO is examined using the Niño 3.4 index and the El Niño Modoki Index (EMI)^33^. The Niño 3.4 index is the area-averaged SST anomaly over the Niño 3.4 region (5°S-5°N, 190–240°E). The EMI is used to detect the ENSO events that have the maximum SST anomaly near the dateline and is the difference between the area-averaged central Pacific SSTs (10°S–10°N, 165–220°E) and the sum of the half of the area-averaged eastern Pacific SSTs (15°S–5°N, 250–290°E) and western Pacific SSTs (10°S–20°N, 125–145°E).

The IOD is monitored by the Indian Ocean Dipole mode index (DMI)^3^, which is the difference of the area-averaged SST anomalies in the western pole (10°S–10°N, 50–70°E) and the eastern pole (10°S-eq, 90–110°E).

The SSTs north of Australia (SSTnAU) was defined by the areal-mean SSTs over the domain of 10°S-equator and 110–160°E, following the definition of Hendon et al.^13^.

The SAM index was obtained following Gong and Wang^9^'s definition, which is the difference of normalised zonal-mean mean sea level pressure anomalies between 40°S and 65°S.

The Real-Time Multivariate MJO Index (RMM) consists of the expansion coefficients of the first two leading modes of empirical orthogonal functions (EOFs) of the combined fields of daily 850 and 200 hPa zonal winds and satellite-observed outgoing long wave radiation data over 15°S–15°N^40^ that capture the amplitude and the eastward propagation of the MJO. The RMM data and comprehensive information about the MJO are available at http://www.bom.gov.au/climate/mjo/.

### Statistical synthesis using multiple linear regression

The synthesis (or reconstruction) of the Australian rainfall anomalies of spring 2020, derived from multiple linear regression analysis (Fig. 3), are computed as follows:

First, the multiple linear regression coefficients are obtained for the training period 1979–2019:

$$\hat{y}_{t} = \sum b_{i} x_{i,t}$$

where *t* and *i* denote the training period and the number of predictors, respectively; $\hat{y}_{t}$ is the synthesis of the predictand* y* for the training period; *b*_*i*_ is the regression coefficient of the *i-*^th^ predictor, derived from least squares fit regression; and *x*_*i,t*_ is the time series of the *i*-^th^ predictor in the training period.

Then, the synthesis for 2020 is obtained by plugging the predictor values of 2020 in the multiple linear regression model:

$$\hat{y}_{2020} = \sum b_{i} x_{i,2020}$$

### Statistical significance test

Statistical significance on correlation and regression was tested by a two-tailed Student t-test with 41 samples of 1979–2019 data. Statistical significance on the difference of two means of high vs low MJO812 cases was also tested by a two-tailed Student t-test but with six samples for high MJO812 and eight samples for low MJO812 events.

### ACCESS-S1 system

ACCESS-S1 is the Australian Bureau of Meteorology (BoM)'s dynamical sub-seasonal to seasonal climate forecast system^18^, which is based on the UKMO GloSea5 system^58^. The atmosphere is resolved on a ~ 60 km grid with 85 vertical levels, appropriately resolving the stratosphere. The ocean is resolved at 25 km with 75 vertical levels. The atmosphere, land and ocean component models are coupled every three hours.

The real-time forecasts using the operational system for September–November 2020 were initialised with the atmospheric conditions from the BoM's numerical weather prediction system and the ocean conditions provided from the Met Office Forecast Ocean Assimilation Model (FOAM)^59^. The real-time system produces a 33-member and a 11-member ensemble of multi-week and seasonal forecasts every day, respectively. Generation of forecast products provided by the BoM Climate Service uses a lagged ensemble approach to form a 99-member ensemble (9 consecutive days for the seasonal forecast products). For example, forecasts initialised on 1 September 2020 displayed in Figs. 4 and 5 use a 99-member ensemble, consisting of the 11-member forecasts from each day from 24 August to 1 September 2020.

Real-time forecast anomalies were computed against the hindcast climatology over 1990–2012. 11-member hindcasts of ACCESS-S1 out to 6-month lead time are available at four different initialisation dates per month (1^st^, 9^th^, 17^th^ and 25^th^). The zonal and meridional winds, temperatures, humidity, surface pressure, and soil temperatures were initialised using the European Centre for Medium-Range Forecasts Interim Reanalysis (ERA-Interim) data^60^, while the model soil moisture was initialised with the climatology of ERA-Interim computed over 1990–2012^58^. The ocean was initialised with the analysis from FOAM. The 11-member ensemble was produced by perturbing the atmospheric initial conditions^18,61^ and through the Stochastic Kinetic Energy Backscatter scheme (SKEB2)^62^. To compute anomalies of real-time forecasts initialised over 9 consecutive days, we used the climatology of the immediate prior hindcast date for each real-time forecast initialisation date. For example, an anomaly of the real-time forecast initialised on 30 August 2020 was computed relative to the climatology of hindcasts initialised on 25 August for 1990–2012.

For the analysis of the November forecasts, 33-member burst ensemble forecasts (i.e., all initialised on the same date) were used. Further details of the ACCESS-S1 model configuration, initialisation, ensemble generation and forecast performance can be found in Hudson et al. studies^18,61^.

## Supplementary Information

Supplementary Information.

## Data Availability

Reynolds OI SST analysis set is available at https://psl.noaa.gov/data/gridded/data.noaa.oisst.v2.html. Hurrell et al. (2008) SST analysis set is available at https://climatedataguide.ucar.edu/climate-data/merged-hadley-noaaoi-sea-surface-temperature-sea-ice-concentration-hurrell-et-al-2008. NOAA interpolated OLR dataset is available at https://psl.noaa.gov/data/gridded/data.interp_OLR.html. JRA-55 set is available at https://rda.ucar.edu/datasets/ds628. AWAP rainfall data set is available at http://www.bom.gov.au/climate/maps/rainfall/?variable=rainfall&map=totals&period=week&region=nat&year=2021&month=03&day=29.

## References

[1] Zhao M, Hendon HH (2009). Representation and prediction of the Indian Ocean dipole in the POAMA seasonal forecast model. Q. J. R. Meteorol. Soc..

[2] Santoso A, Mcphaden MJ, Cai W (2017). The defining characteristics of ENSO extremes and the strong 2015/2016 El Niño. Rev. Geophys..

[3] Saji NH, Goswami BN, Vinayachandran PN, Yamagata T (1999). A dipole mode in the tropical Indian Ocean. Nature.

[4] Cai W (2011). Teleconnection pathways of ENSO and the IOD and the mechanisms for impacts on Australian rainfall. J. Clim..

[5] Lim E-P, Hendon HH (2017). Causes and predictability of the negative Indian Ocean dipole and its impact on La Niña during 2016. Sci. Rep..

[6] Hio Y, Yoden S (2005). Interannual variations of the seasonal march in the Southern Hemisphere stratosphere for 1979–2002 and characterization of the unprecedented Year 2002. J. Atmos. Sci..

[7] Byrne NJ, Shepherd TG (2018). Seasonal persistence of circulation anomalies in the Southern Hemisphere stratosphere and its implications for the troposphere. J. Clim..

[8] Lim E-P, Hendon HH, Thompson DWJ (2018). Seasonal evolution of stratosphere-troposphere coupling in the Southern Hemisphere and implications for the predictability of surface climate. J. Geophys. Res. Atmos..

[9] Gong D, Wang S (1999). Definition of Antarctic Oscillation index. Geophys. Res. Lett..

[10] Thompson DWJ, Wallace JM (2000). Annular Mode in the extratropical circulation. Part I: Month-to-month variability. J. Clim..

[11] Lim E-P, Hendon HH, Rashid H (2013). Seasonal predictability of the Southern Annular Mode due to its association with ENSO. J. Clim..

[12] Seviour WJM (2014). Skillful seasonal prediction of the Southern Annular Mode and Antarctic ozone. J. Clim..

[13] Hendon HH, Lim E-P, Arblaster JM, Anderson DLT (2014). Causes and predictability of the record wet east Australian spring 2010. Clim. Dyn..

[14] Lim E-P (2021). Tropical forcing of Australian extreme low minimum temperatures in September 2019. Clim. Dyn..

[15] Lim E-P (2021). The 2019 Southern Hemisphere stratospheric polar vortex weakening and its impacts. Bull. Am. Meteorol. Soc..

[16] Nguyen H, Wheeler MC, Hendon HH, Lim E-P, Otkin JA (2021). The 2019 flash droughts in subtropical eastern Australia and their association with large-scale climate drivers. Weather Clim. Extrem..

[17] BoM. Annual climate statement 2019. Available at: http://www.bom.gov.au/climate/current/annual/aus/2019/#:~:text=2019 was Australia’s warmest year,1.33 °C in 2013.&text=Warming associated with anthropogenic climate,over one degree since 1910, (2020).

[18] Hudson D (2017). ACCESS-S1: The new Bureau of Meteorology multi-week to seasonal prediction system. J. South. Hemisph. Earth Syst. Sci..

[19] Hendon, H. H. & Lim, E.-P. Long lead prediction of the 2019 climate extremes. in *the Australian Meteorological and Oceanographic Society Annual Conference Abstracts* p 269, https://drive.google.com/file/d/13uu4XtHKcWKcSTZf2aKci_gNA0HjTikd/view, (2021).

[20] Hudson DA (2017). ACCESS-S1 The new Bureau of Meteorology multi-week to seasonal prediction system. J. South. Hemisph. Earth Syst. Sci..

[21] King AD (2020). Sub-seasonal to seasonal prediction of rainfall extremes in Australia. Q. J. R. Metrol. Soc..

[22] Hendon HH, Lim E-P, Abhik S (2020). Impact of interannual ozone variations on the downward coupling of the 2002 Southern Hemisphere stratospheric warming. J. Geophys. Res. Atmos..

[23] Marshall AG, Hendon HH, Hudson D (2021). Influence of the Madden-Julian Oscillation on multiweek prediction of Australian rainfall extremes using the ACCESS-S1 prediction system. J. South. Hemisph. Earth Syst. Sci..

[24] Marshall AG, Gregory PA, de Burgh-Day CO, Griffiths M (2021). Subseasonal drivers of extreme fire weather in Australia and its prediction in ACCESS-S1 during spring and summer. Clim. Dyn..

[25] L’Heureux ML, Thompson DWJ (2006). Observed relationships between the El Niño-Southern Oscillation and the extratropical zonal-mean circulation. J. Clim..

[26] Lim E-P, Hendon HH (2015). Understanding and predicting the strong Southern Annular Mode and its impact on the record wet east Australian spring 2010. Clim. Dyn..

[27] Risbey JS, Pook MJ, Wheeler MC, Hendon HH (2009). On the remote drivers of rainfall variability in Australia. Mon. Weather Rev..

[28] Reynolds RW, Rayner NA, Smith TM, Stokes DC, Wang W (2002). An improved in situ and satellite SST analysis for climate. J. Clim..

[29] Hurrell JW, Hack JJ, Shea D, Caron JM, Rosinski J (2008). A new sea surface temperature and sea ice boundary dataset for the community atmosphere model. J. Clim..

[30] Liebmann B, Smith CA (1996). Description of a complete (interpolated) outgoing longwave radiation dataset. Bull. Am. Meteorol. Soc..

[31] Kobayashi S (2015). The JRA-55 reanalysis: General specifications and basic characteristics. J. Meteorol. Soc. Japan. Ser..

[32] Jones D, Wang W, Fawcett R (2009). High-quality spatial climate data-sets for Australia. Aust. Meteorol. Oceanogr. J..

[33] Ashok K, Behera SK, Rao SA, Weng H, Yamagata TE (2007). Niño Modoki and its possible teleconnection. J. Geophys. Res..

[34] Deser C, Phillips AS, Alexander MA (2010). Twentieth century tropical sea surface temperature trends revisited. Geophys. Res. Lett..

[35] Lim EP (2016). Interaction of the recent 50 year SST trend and La Niña 2010: Amplification of the Southern Annular Mode and Australian springtime rainfall. Clim. Dyn..

[36] Ummenhofer CC (2009). What causes southeast Australia’s worst droughts?. Geophys. Res. Lett..

[37] Abram NJ (2020). Coupling of Indo-Pacific climate variability over the last millennium. Nature.

[38] Madden RA, Julian PR (1971). Detection of a 40–50 day oscillation in the zonal wind in the tropical pacific. J. Atmos. Sci..

[39] Wheeler MC, Hendon HH, Cleland S, Meinke H, Donald A (2009). Impacts of the Madden–Julian oscillation on Australian rainfall and circulation. J. Clim..

[40] Wheeler MC, Hendon HH (2004). An All-season real-time multivariate MJO index: Development of an index for monitoring and prediction. Mon. Weather Rev..

[41] Student. The probable error of a mean. *Biometrika* **6**, 1 (1908).

[42] Marshall AG, Hendon HH (2019). Multi-week prediction of the Madden–Julian oscillation with ACCESS-S1. Clim. Dyn..

[43] McBride JL, Nicholls N (1983). Seasonal relationships between Australian rainfall and the Southern oscillation. Mon. Weather Rev..

[44] Power S, Casey T, Folland C, Colman A, Mehta V (1999). Inter-decadal modulation of the impact of ENSO on Australia. Clim. Dyn..

[45] Holgate, C. M., Van Dijk, A. I. J. M., Evans, J. P. & Pitman, A. J. Local and remote drivers of Southeast Australian drought. *Geophys. Res. Lett.* **47**, (2020).

[46] King AD, Pitman AJ, Henley BJ, Ukkola AM, Brown JR (2020). The role of climate variability in Australian drought. Nat. Clim. Chang..

[47] Luo J-J, Liu G, Hendon H, Alves O, Yamagata T (2017). Inter-basin sources for two-year predictability of the multi-year La Niña event in 2010–2012. Sci. Rep..

[48] Battisti DS, Hirst AC (1989). Interannual variability in a tropical atmosphere-ocean model: Influence of the basic state, ocean geometry and nonlinearity. J. Atmos. Sci..

[49] Jin F, An S (1999). Within the equatorial ocean recharge oscillator model for ENSO. Geophys. Res. Lett..

[50] Nicholls N (1984). The Southern Oscillation, sea-surface-temperature, and interannual fluctuations in Australian tropical cyclone activity. J. Climatol..

[51] van Rensch P (2019). Mechanisms causing east Australian spring rainfall differences between three strong El Niño events. Clim. Dyn..

[52] Meyers G, McIntosh P, Pigot L, Pook M (2007). The years of El Niño, La Niña and interactions with the tropical Indian Ocean. J. Clim..

[53] Zhu H, Maloney E, Hendon H, Stratton R (2017). Effects of the changing heating profile associated with melting layers in a climate model. Q. J. R. Meteorol. Soc..

[54] Cottrill A (2013). Seasonal forecasting in the pacific using the coupled model POAMA-2. Weather Forecast..

[55] Zhao M, Hendon H, Oscar A, Liu G, Guomin W (2016). Weakened Eastern Pacific El Niño predictability in the early twenty-first century. J. Clim..

[56] Lim E-P, Hendon HH, Zhao M, Yin Y (2017). Inter-decadal variations in the linkages between ENSO, the IOD and south-eastern Australian springtime rainfall in the past 30 years. Clim. Dyn..

[57] Grose, M. R. *et al.* Insights from CMIP6 for Australia’s future climate. *Earth Futur.* **8**, e2019EF001469 (2020).

[58] MacLachlan C (2015). Global Seasonal forecast system version 5 (GloSea5): A high-resolution seasonal forecast system. Q. J. R. Meteorol. Soc..

[59] Waters J (2015). Implementing a variational data assimila- tion system in an operational 1/4 degree global ocean model. Q. J. R. Meteorol. Soc..

[60] Dee D (2011). The ERA-Interim reanalysis: Configuration and performance of the data assimilation system. Q. J. R. Meteorol. Soc..

[61] Hudson D (2020). Corrigendum to: ACCESS-S1: The new Bureau of Meteorology multi-week to seasonal prediction system. J. South. Hemisph. Earth Syst. Sci..

[62] Bowler NE, Arribas A, Beare SE, Mylne KR, Shutts GJ (2009). The local ETKF and SKEB: Upgrades to the MOGREPS short-range ensemble prediction system. Q. J. R. Meteorol. Soc..

[63] Henley BJ (2015). A tripole index for the interdecadal Pacific Oscillation. Clim. Dyn..

