# Supplementary material for: Why Australia was not wet during spring 2020 despite La Niña
Source: Sci Rep. 2021 Sep 16;11:18423. doi: 10.1038/s41598-021-97690-w (PMC8445951; doi:10.1038/s41598-021-97690-w)
Supplement: Supplementary file 1 — Supplementary Information. [file 41598_2021_97690_MOESM1_ESM.pdf]

Supplementary Information

Why Australia was Not Wet during Spring 2020 despite La Niña

Eun-Pa Lim[1], Debra Hudson[1], Andrew G. Marshall[1], Matthew C. Wheeler[1], Andrew King[2], Hongyan Zhu[1], Harry H. Hendon[1,3], Catherine de Burgh-Day[1], Blair Trewin[1], Morwenna Griffiths[1], Avijeet Ramchurn[1], and Griffith Young[1]

[1] Bureau of Meteorology, Melbourne, VIC, Australia

[2] School of Geography, Earth, and Atmospheric Sciences and ARC Centre of Excellence for Climate Extremes, University of Melbourne, Parkville, VIC, Australia

[3] School of Earth Atmosphere and Environment, Monash University, Clayton, VIC, Australia

Corresponding author: Eun-Pa Lim

eun-pa.lim@bom.gov.au

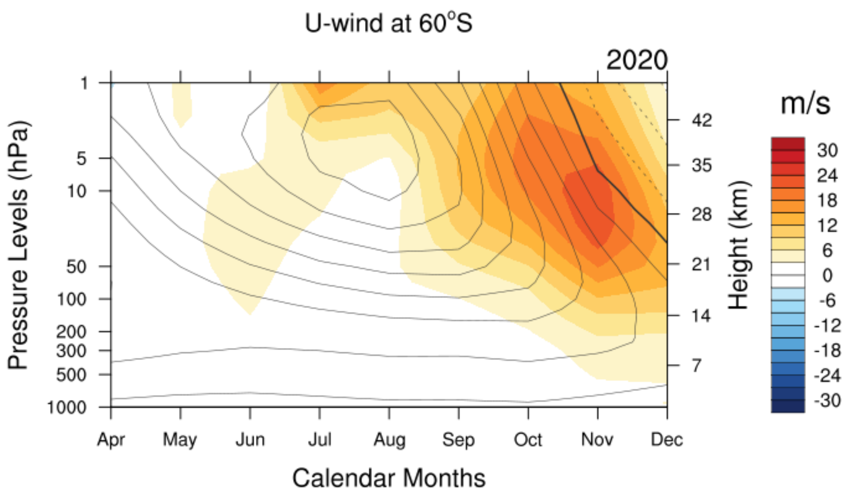

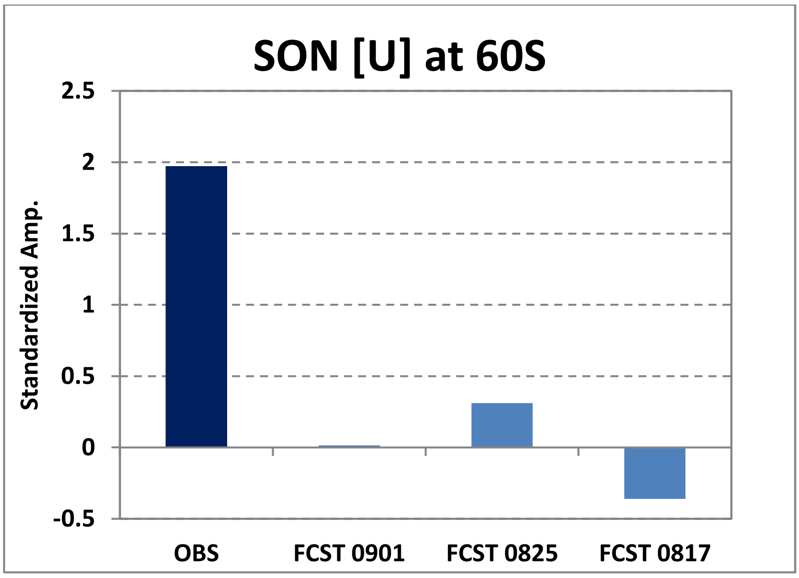

Supplementary Fig S1. **Southern Hemisphere stratospheric polar vortex strengthening of 2020 and its downward coupling.** (a) Zonal-mean zonal wind anomalies (colour shading) at 60°S as a function of height and calendar months overlaid with the monthly climatological winds (contours). The contour interval is 10 m/s. Zero contour is thick, and negative contours are dashed. JRA-55 data[1] are used for this observational analysis. (b) Standardized amplitudes of the anomalous springtime Antarctic stratospheric polar vortex (September-November mean zonal-mean zonal winds at 60°S and at 10 hPa) of 2020 in the observational analysis (dark blue bar) and ACCESS-S1[2] forecasts initialised on 1 September, 25 August, and 17 August of 2020 (light blue bars). The climatology and standard deviation of the observed and forecast data are from the period 1990-2012 when ACCESS-S1 hindcasts are available. Details of ACCESS-S1 are provided in the Methods section of the main article and also in Hudson et al.[2]

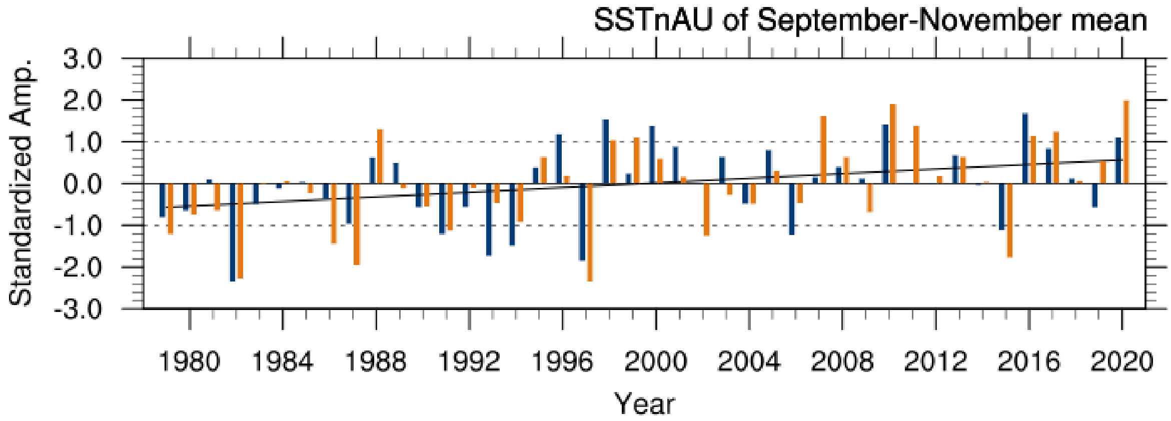

Supplementary Fig S2. Time series of the sea surface temperatures (SSTs) north of Australia (10°S-equator, 110-160°E). The blue bars show the raw data, and the black line indicates a linear trend in the raw data. The orange bars show the predicted SSTs north of Australia (SSTnAU) using the de-trended Niño3.4 index of September-November mean and the trend (i.e., time), which was obtained with cross-validation (i.e., building the multiple linear regression model 42 times, leaving a year out each time to predict the year left out through 1979-2020). The thin dashed horizontal lines indicate ±1 standard deviation. The raw and the predicted times series are highly correlated at 0.76 over 1979-2020, and the raw time series is correlated with the de-trended Niño3.4 SST index and with the trend at -0.71 and 0.38, respectively, for the same 42 years.

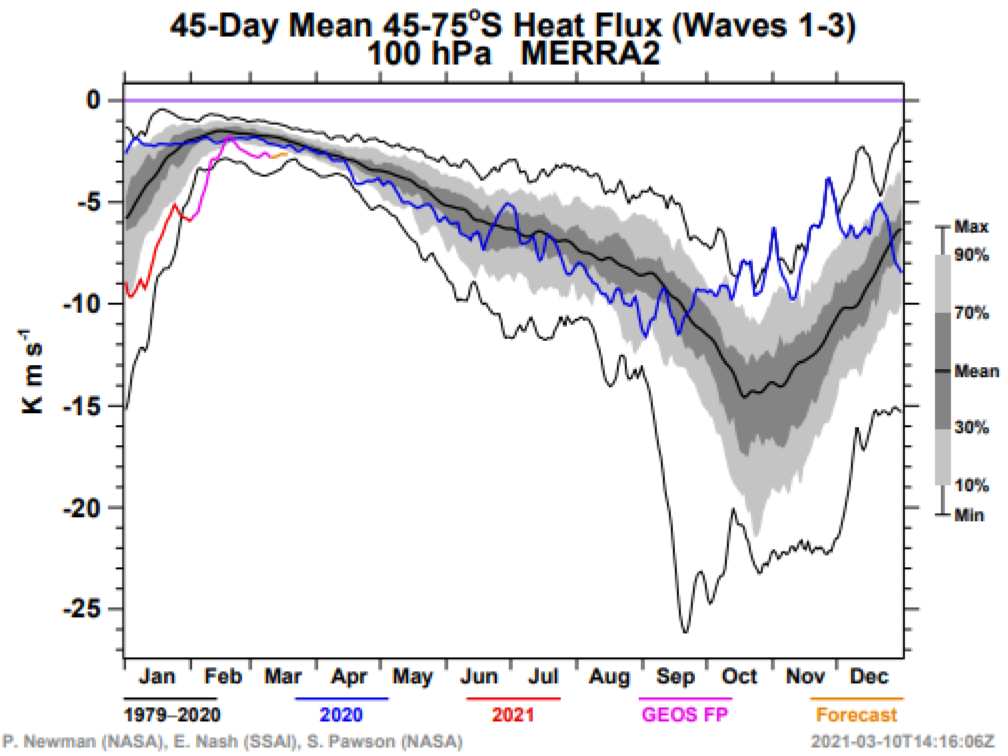

Supplementary Fig S3. 45-day mean heat flux by planetary-scale waves (wavenumber 1-3) averaged over 45-75°S at the 100 hPa level. The blue line indicates the 2020 heat flux, the thin black lines indicate the maximum and minimum values of the heat flux since 1979, the thick black line denotes the climatological mean; the dark grey shading indicates 30-70% of the historical distribution of the Modern-Era Retrospective analysis for Research and Applications, Version 2[3]; and the light grey shading indicates 10-30% and 70-90% of the distribution. The data of GEOS FP are analyses and forecasts from the Goddard Earth Observing System Forward Processing assimilation system produced in real-time (https://gmao.gsfc.nasa.gov/GMAO_products/). The plot was taken from the NASA Ozone Watch page (https://ozonewatch.gsfc.nasa.gov/meteorology/figures/merra2/heat_flux/vt1-3w45_75-45s_100_2021_merra2.pdf).

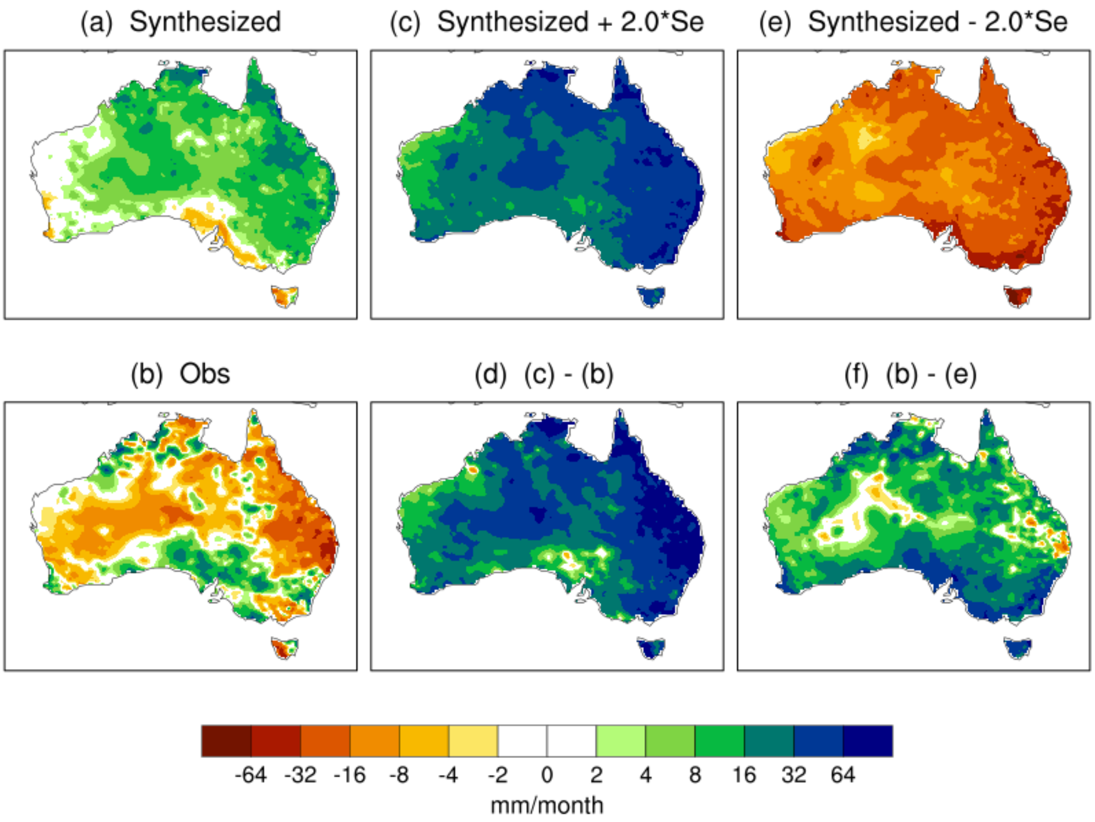

Supplementary Fig S4. Statistical prediction of September to November mean rainfall of 2020. (a) Synthesized (predicted) rainfall by the multiple linear regression model using the de-trended Niño3.4 SST, the de-trended DMI, the de-trended SSTnAU, the de-trended SAM, and the linear trend (i.e., time) since 1979 as predictors. The training period was 1979-2019. (b) Observed springtime rainfall anomalies of 2020. (a) and (b) are the same plots as Figures 3f and g in the main article. (c) The upper boundary of the 95% prediction interval of the statistical model built with 1979-2019 data. It was obtained by the prediction of 2020 plus the t-value (which is 2.02 for the sample size of 40) multiplied by the standard error estimated over 1979-2019. (d) Difference between the upper boundary estimate of the statistical model and the observed rainfall anomaly for spring 2020. (e) The lower boundary of the 95% prediction interval, which was obtained by the prediction of 2020 minus the t-value multiplied by the standard error. (f) Difference between the observed rainfall anomaly and the lower boundary estimate of the statistical model for spring 2020. In (d) and (f) the green to blue colour shading indicates that the observation was below the upper boundary and above the lower boundary of the 95% prediction interval, respectively, whereas the yellow to orange colour shading indicates that the observation was outside of the 95% prediction interval. Thus, the dryness over south-eastern Queensland and central-western Australia was unpredictable by this model even with the consideration of the uncertainty. Maps were generated using the NCAR Command Language version 6.6.2 (www.ncl.ucar.edu).

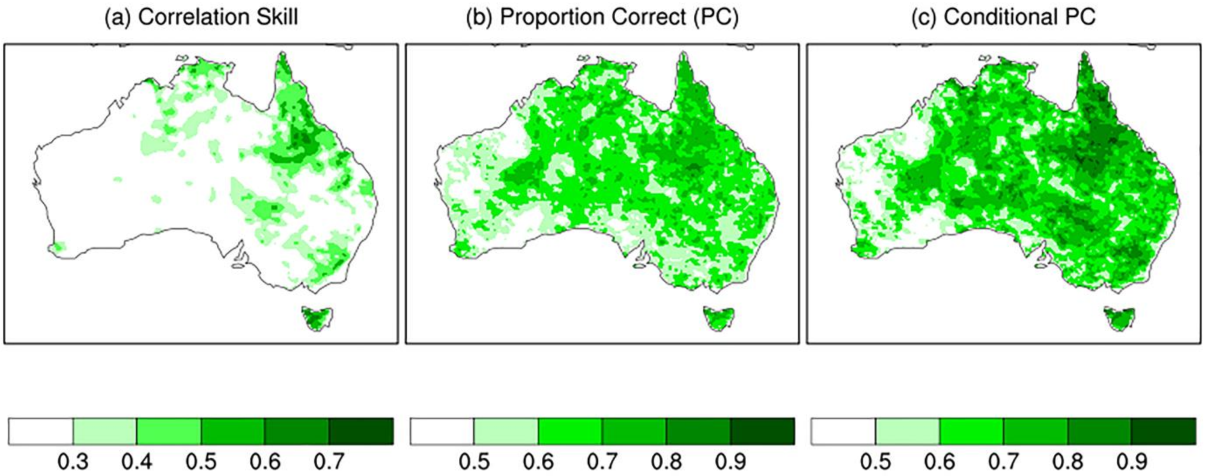

Supplementary Fig S5. (a) Correlation skill of the statistical forecast model, which was assessed through a cross-validation process by leaving a year out, constructing the model with the rest of the data, making a prediction for the year left out, and verifying the forecast throughout the period 1979-2020. Correlation skill greater than 0.31 is statistically significant at the 5% level with 42 samples. (b) Proportion correct[4] for predicting above/below average. (c) Proportion correct conditioned by prediction being greater than $|0.5\sigma|$. Together with Supplementary Fig. S4, Fig. S5 suggests that although the standard error of this model is large, it predicts the sign of the observed rainfall anomalies significantly better than random guessing (i.e., proportion correct 0.5 for above/below average) as demonstrated in (b), and this is certainly the case for the rainfall forecasts with moderate to strong amplitudes (i.e., detectable magnitude of signal) as demonstrated in (c). Thus, the false alarm like 2020 is not a common case of this model. Maps were generated using the NCAR Command Language version 6.6.2 (www.ncl.ucar.edu).

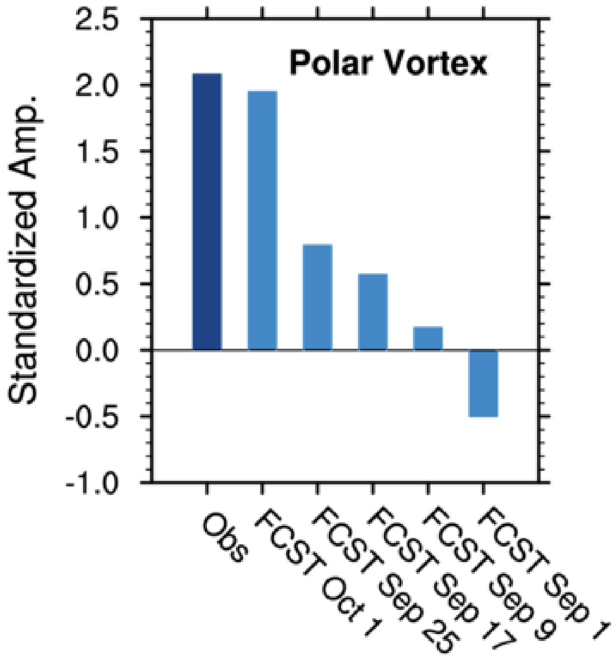

Supplementary Fig S6. Observed and forecast Antarctic stratospheric polar vortex of the October to November mean of 2020. The polar vortex strength was measured by the zonal-mean zonal wind anomaly of the October to November mean at 60°S and at the 10 hPa level. The light blue colour bars show the ACCESS-S1 forecasts initialised on different dates from 1 September to 1 October 2020, and the dark blue bar shows the observed amplitude. The observed and forecast anomalies were relative to their respective climatologies of 1990-2012 and were normalized by their respective standard deviations of the climatological period.

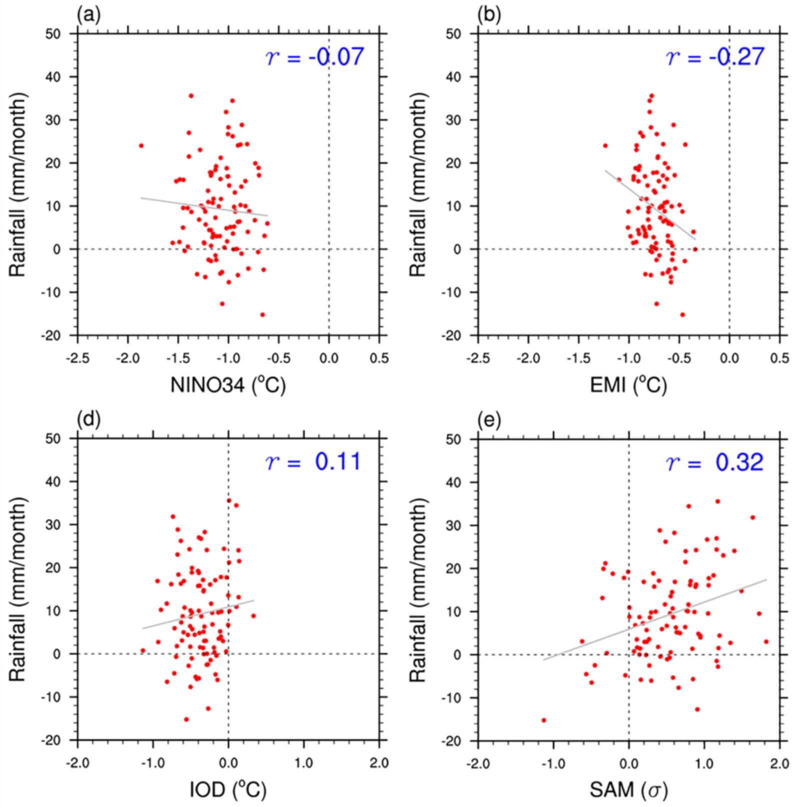

Supplementary Fig S7. Relationship of eastern Australian rainfall and the climate indices in ACCESS-S1 forecasts for spring 2020. Displayed are 99 ensemble member forecasts of September-November mean eastern Australian rainfall (averaged over the land areas east of 140°E) versus their forecasts of (a) the Niño3.4 SSTs, (b) El Niño Modoki (EMI), (c) SSTs north of Australia, (d) the Indian Ocean Dipole mode (IOD), and (e) the Southern Annular Mode (SAM). Forecasts were initialised on 1 September 2020. The blue coloured number in each panel is the correlation coefficient between the forecast rainfall and the forecast climate index.

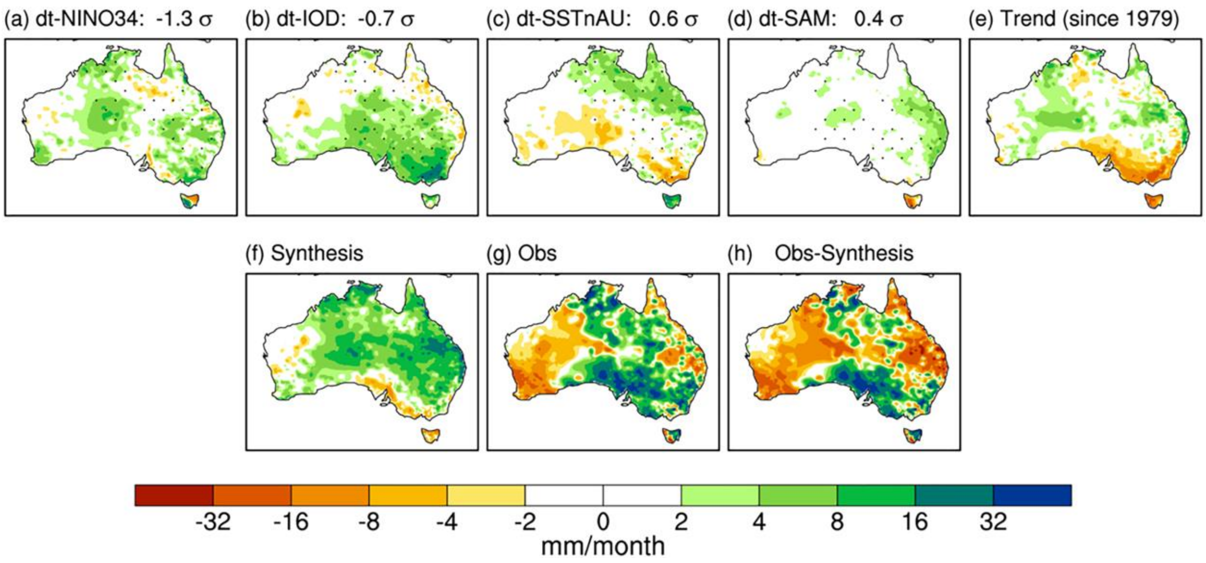

Supplementary Fig S8. Synthesis of September-October mean rainfall anomalies of 2020 using multiple linear regression onto key climate indices. The contributions from the individual predictors are (a) de-trended Niño3.4 SST index, (b) de-trended DMI, (c) de-trended SSTs north of Australia (eq-10°S,110-160°E), (d) de-trended SAM, and (e) time (i.e., trend). The full synthesis using all five predictors is shown in (f). (g) Observed 2020 September-October mean rainfall anomaly relative to the climatology of 1990-2012. (h) Difference between the observed and the synthesized rainfall. In (a)-(e) synthesis anomalies were computed by the regression coefficients obtained over 1979-2019 and then scaled by the predictor values of 2020 (see the Methods section in the main article). Stippling in (a)-(e) indicates where correlation between the rainfall and each predictor is statistically significant over 1979-2019 at the 5% level. Maps were generated using the NCAR Command Language version 6.6.2 (www.ncl.ucar.edu).

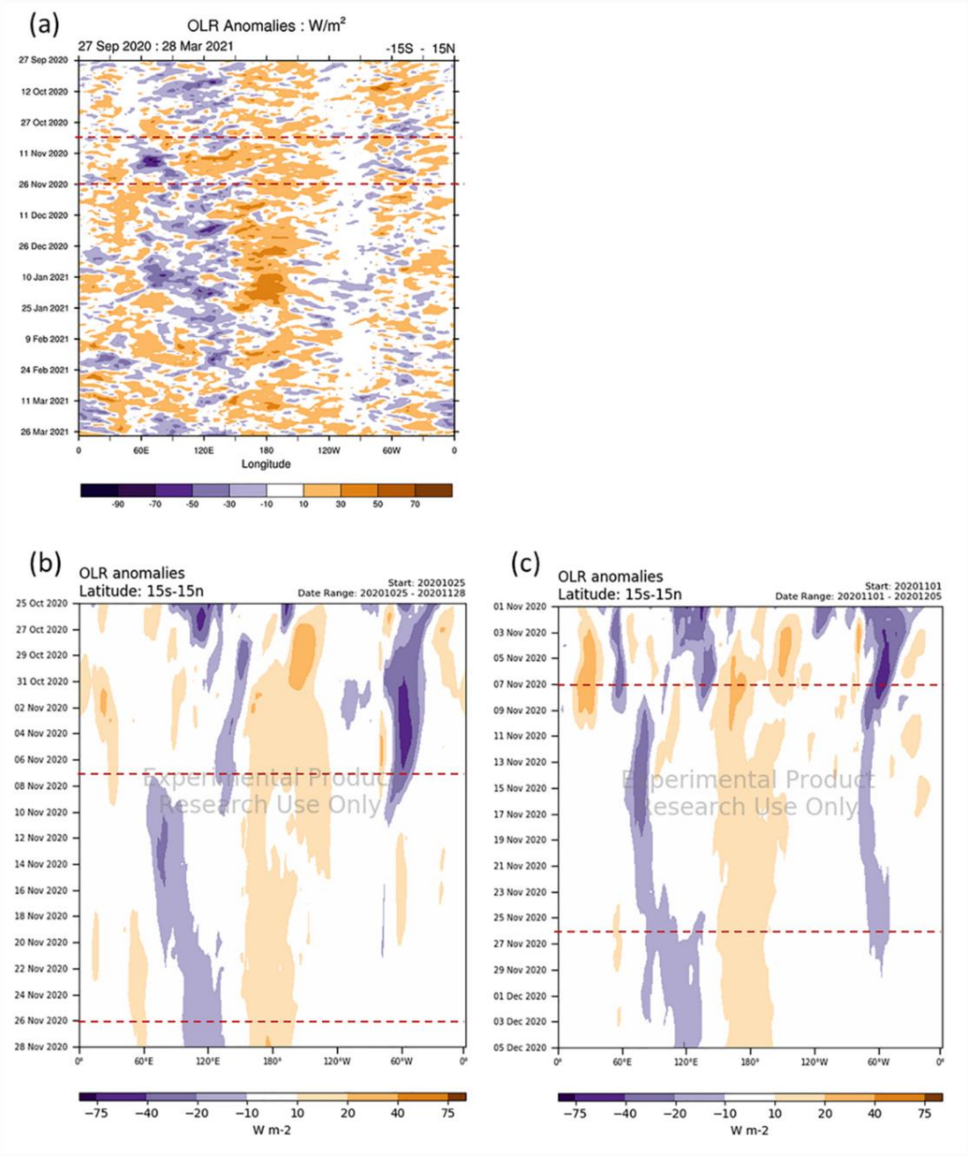

Supplementary Fig S9. Observed and forecast temporal and spatial evolution of the outgoing long wave radiation (OLR) associated with the Madden-Julian Oscillation (MJO) occurred in November 2020. (a) Observed analysis from 27 September 2020 to 28 March 2021 was taken from http://www.bom.gov.au/climate/mjo/#tabs=Time-longitude, and (b), (c) ACCESS-S1 forecasts initialised on 25 October and 1 November 2020, respectively. The red horizontal dashed lines indicate the period between 7-26 November when MJO812 was observed. In (a) the MJO activity over the equatorial Indian Ocean propagated to the east in that period, which resulted in the extension of the area of suppressed convection by La Niña to the west (i.e., 90°E). In contrast, as shown in (b) and (c), ACCESS-S1 did not simulate the MJO related suppressed convection anomalies, which was represented as small amplitudes of the MJO in Phases 1 and 2 (main Figure 11). Panel (a) was generated by Fortran 90 and NCAR Graphics (http://ngwww.ucar.edu/), and panels (b) and (c) were generated by Python version 3.6.1 (https://www.python.org/).

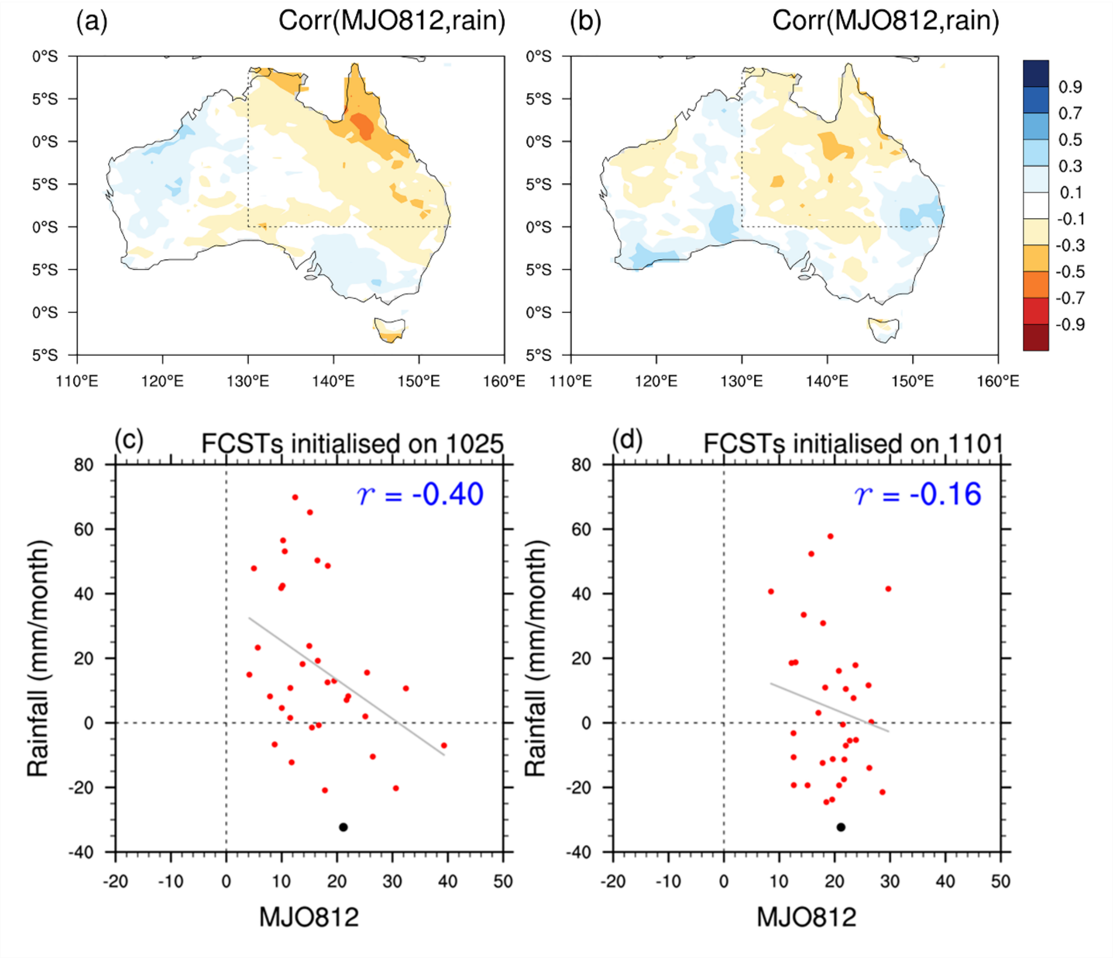

Supplementary Fig S10. Relationship of November rainfall with the MJO812 in the 33 member forecasts initialised on (a,c) 25 October 2020 and (b,d) 1 November 2020. In (a) and (b) correlation was computed for rainfall at each grid point with the raw MJO812 index (i.e., without removing the ENSO-related component). For (c) and (d), forecast rainfall was averaged over the central to eastern Australia north of 30°S (the area indicated by the thin dashed lines in (a) and (b)) and was plotted against the forecast MJO amplitude. Correlation coefficients are shown in the top right corners. The negative correlation indicates that the higher the forecast MJO812 amplitudes are, the less the forecast rain falls. The black solid dot indicates the observed MJO812 and central-eastern Australian rainfall values. While the ensemble mean forecasts produce weak MJO812 for November 2020 as displayed in Figure 12g, 33 individual forecasts encompass the observed MJO magnitude in (c) and (d). This suggests that each forecast has a large MJO amplification in Phases 8, 1 *or* 2 sometime in November 2020 although there is a large spread in the forecast ensemble for Phases 1 and 2 in mid- to late November. Forecasts initialised on 25 October 2020 tend to better capture the MJO-rainfall relationship than those initialised on 1 November 2020. Maps were generated using the NCAR Command Language version 6.6.2 (www.ncl.ucar.edu).

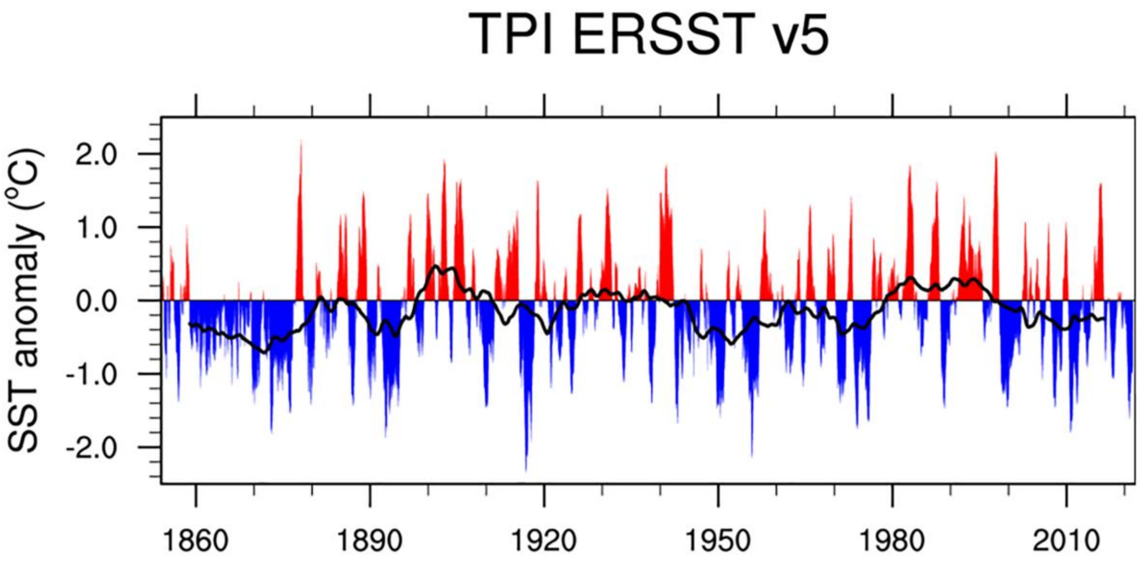

Supplementary Fig S11. Henley et al[6]'s Tripole Index that monitors the Inter-decadal Pacific Oscillation (IPO)[5,6]. The ERSST version 5 unfiltered tripole index was obtained from https://psl.noaa.gov/data/timeseries/IPOTPI/. It is a monthly index (colour-filled line) formed by the difference between the SST anomalies averaged over the central equatorial Pacific and those averaged in the Northwest and Southwest Pacific. The IPO is depicted by the low-frequency filtered index (thick black line), being computed using a 10-year running mean of the monthly index. The timeseries of the IPO index shown here bears a strong resemblance to that calculated using HadISST version 1 for years after 1940 (not shown).

References

1. Kobayashi, S. *et al.* The JRA-55 Reanalysis: General Specifications and Basic Characteristics. *J. Meteorol. Soc. Japan. Ser. II* **93**, 5–48 (2015).

2. Hudson, D. A. *et al.* ACCESS-S1 The new Bureau of Meteorology multi-week to seasonal prediction system. *J. South. Hemisph. Earth Syst. Sci.* **67**, 132–159 (2017).

3. Gelaro, R. *et al.* The Modern-Era Retrospective Analysis for Research and Applications, Version 2 (MERRA-2). *J. Clim.* **30**, 5419–5454 (2017).

4. Wilks, D. S. *Statistical methods in the atmospheric sciences*. (Academic Press, Inc., 2006).

5. Power, S., Casey, T., Folland, C., Colman, A. & Mehta, V. Inter-decadal modulation of the impact of ENSO on Australia. *Clim. Dyn.* **15**, 319–324 (1999).

6. Henley, B. J. *et al.* A Tripole Index for the Interdecadal Pacific Oscillation. *Clim. Dyn.* **45**, 3077–3090 (2015).
